# 2D Materials in Advanced Electronic Biosensors for Point‐of‐Care Devices

**DOI:** 10.1002/advs.202401386

**Published:** 2024-06-18

**Authors:** Sobia Nisar, Ghulam Dastgeer, Zafar Muhammad Shazad, Muhammad Wajid Zulfiqar, Amir Rasheed, Muhammad Zahir Iqbal, Kashif Hussain, Iqra Rabani, Deok‐kee Kim, Ahmad Irfan, Aijaz Rasool Chaudhry

**Affiliations:** ^1^ Department of Electrical Engineering Sejong University Seoul 05006 Republic of Korea; ^2^ Department of Convergence Engineering for Intelligent Drone Sejong University Seoul 05006 Republic of Korea; ^3^ Department of Physics & Astronomy Sejong University Seoul 05006 Republic of Korea; ^4^ SKKU Advanced Institute of Nanotechnology (SAINT) Sungkyunkwan University Suwon 16419 Republic of Korea; ^5^ Department of Chemical Polymer and Composite Engineering University of Engineering & Technology Faisalabad Campus Lahore 38000 Pakistan; ^6^ Department of Semiconductor Engineering Sejong University Seoul 05006 Republic of Korea; ^7^ School of Materials Science and Engineering Anhui University Hefei Anhui 230601 China; ^8^ Renewable Energy Research Laboratory Faculty of Engineering Sciences Ghulam Ishaq Khan Institute of Engineering Sciences and Technology Topi Khyber Pakhtunkhwa 23640 Pakistan; ^9^ THz Technical Research Center; Shenzhen Key Laboratory of Micro‐Nano Photonic Information Technology; Key Laboratory of Optoelectronic Devices and Systems, College of Physics and Optoelectronic Engineering Shenzhen University Shenzhen Guangdong Province 518060 China; ^10^ School of Materials Science and Engineering CAPT Peking University Beijing 100871 China; ^11^ Department of Nanotechnology and Advanced Materials Engineering Sejong University Seoul 05006 Republic of Korea; ^12^ Department of Chemistry College of Science King Khalid University P. O. Box 9004 Abha 61413 Saudi Arabia; ^13^ Department of Physics College of Science University of Bisha P.O. Box 551 Bisha 61922 Saudi Arabia

**Keywords:** 2D materials, analyte detection, biomedical diagnostics, field‐effect transistor, label‐free detection, point‐of‐care (PoC)

## Abstract

Since two‐dimensionalal (2D) materials have distinct chemical and physical properties, they are widely used in various sectors of modern technologies. In the domain of diagnostic biodevices, particularly for point‐of‐care (PoC) biomedical diagnostics, 2D‐based field‐effect transistor biosensors (bio‐FETs) demonstrate substantial potential. Here, in this review article, the operational mechanisms and detection capabilities of biosensing devices utilizing graphene, transition metal dichalcogenides (TMDCs), black phosphorus, and other 2D materials are addressed in detail. The incorporation of these materials into FET‐based biosensors offers significant advantages, including low detection limits (LOD), real‐time monitoring, label‐free diagnosis, and exceptional selectivity. The review also highlights the diverse applications of these biosensors, ranging from conventional to wearable devices, underscoring the versatility of 2D material‐based FET devices. Additionally, the review provides a comprehensive assessment of the limitations and challenges faced by these devices, along with insights into future prospects and advancements. Notably, a detailed comparison of FET‐based biosensors is tabulated along with various other biosensing platforms and their working mechanisms. Ultimately, this review aims to stimulate further research and innovation in this field while educating the scientific community about the latest advancements in 2D materials‐based biosensors.

## Graphical abstract

This article explores the working mechanism and detecting capacities of FET biosensors made from diverse 2D materials such as graphene, TMDCs, and black phosphorus. These materials enable the sensors to achieve low detection limits, real‐time monitoring, label‐free diagnostics, and great selectivity. It also discusses constraints and future directions, paving the path for advances in biosensor technology.

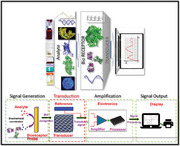

## 1 Introduction

Since the challenges of the COVID‐19 pandemic, the demand for biosensors has surged exponentially, playing a pivotal role in diagnostics, monitoring, and research. The significance of biosensors in the realm of biotechnology lies in their ability to provide rapid, accurate, and scalable detection methods, thereby contributing indispensably to the swift and precise management of infectious diseases, including the unprecedented challenges posed by the ongoing global health crisis.^[^ ^1^, ^2^, ^3^, ^4^ ^]^ Disease mortality can be decreased in large part by receiving early diagnosis of illnesses. Owing to their outstanding sensitivity, fast detection, and outstanding selectivity, FET‐based biosensors have emerged as one of the detection technologies with the most potential for use in point‐of‐care diagnostics as onsite testing kits.^[^ ^5^, ^6^, ^7^ ^]^ Moreover, with the inclusion of vital disciplines consisting of genetic engineering,^[^ ^8^ ^]^ molecular biology,^[^ ^9^ ^]^ cell biology,^[^ ^10^ ^]^ biochemistry,^[^ ^11^ ^]^ immunology,^[^ ^12^ ^]^ and biosensing,^[^ ^13^, ^14^ ^]^ this increase in interest has caused a thorough shift in the field of biotechnology. The integration of biosensors into mechanical devices designed for healthcare monitoring and support represents a pivotal advancement in modern healthcare technology.^[^ ^15^, ^16^ ^]^ Among them, biosensing stands out as a foundational technology, especially in complicated circumstances, as is essential for disease detection, epidemic management, and health testing.^[^ ^17^, ^18^, ^19^, ^20^ ^]^

**Figure 1:**
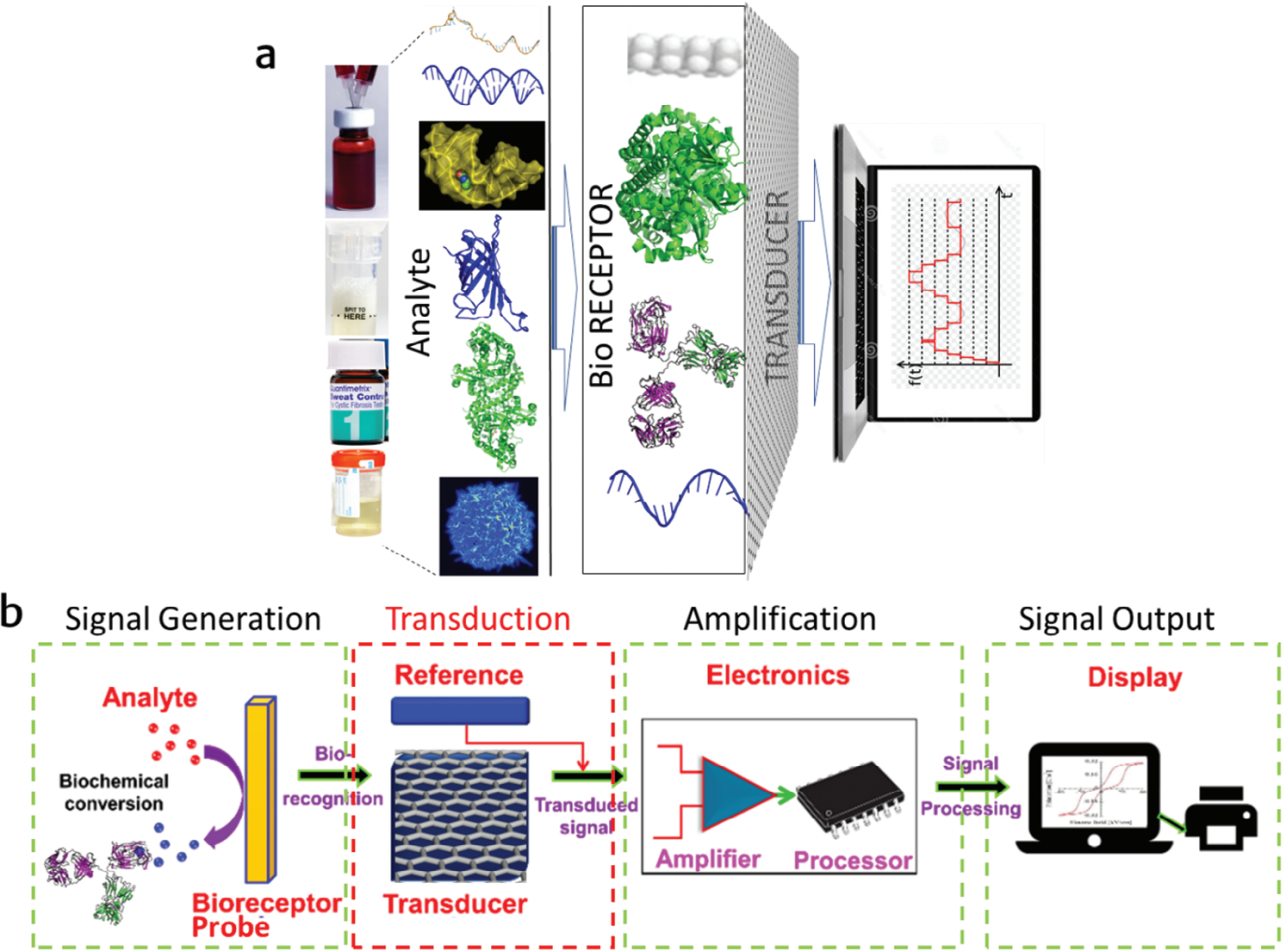
Illustration of the components of the biosensor in a flow chart format. a) Main parts of biosensor: target analyte, bioreceptor‐transducer, electronic system. b) Sequential depiction of the biosensor operation: interaction between the target analyte and bioreceptor, transduction process, signal amplification, and final display of results.

Since Clark and Lyons' seminal work in 1962, when they first suggested using glucose oxidase (GOD) for electrochemically detecting glucose, the field of biosensing has made considerable strides.^[^ ^21^ ^]^ Their innovative work was a significant turning point in the evolution of biosensing technology. In general, the electronic devices used for biosensing applications are specially designed devices that identify and convert biological events into measurable signals. A biosensor comprises three primary components: a biological recognition element (target analyte/sample), a transducer, and electronic elements for signal processing, as depicted in **Figure** **1**a. Meanwhile, the sequential illustration of biosensor operation, depicting the interaction between the target analyte and bioreceptor, followed by the transduction process, signal amplification, and ultimately showcasing the final display of results is depicted in Figure 1b. Depending on their transducers, biosensors can be categorized as mechanical, optical, electrochemical, or electrical sensors. Mechanical sensors identify binding events by monitoring variations in mechanical characteristics, such as stress or strain in a cantilever structure, or modifications in mechanical waves within specific materials.^[^ ^22^ ^]^ Optical sensors employ diverse techniques, such as surface plasmon resonance, Raman scattering, fluorescence, and colorimetry, to identify variations in light signals.^[^ ^23^ ^]^ Electrochemical sensors detect fluctuations in the concentration of the substance being studied by measuring changes in current, potential, or conductivity.^[^ ^24^ ^]^ Electrical sensors, which can be further divided into such categories as field‐effect transistors and bipolar junction transistors are devices that monitor changes in electrical signals brought on by shifts in the substance's concentration.^[^ ^25^ ^]^ There are advantages and disadvantages to each of these sensor types, the selection of a particular method relies on the requirements of the application, the resources at hand, and various other factors.

The field effect transistor (FET) represents a revolutionary advancement in biosensing technology due to its exceptional qualities, such as label‐free detection, high sensitivity, quick reaction, real‐time measuring capability, low running power, and the viability of miniaturization to a portable device.^[^ ^26^ ^]^ In field‐effect transistor based‐biosensors, the immobilization of bio‐receptors takes place on a semiconductor channel or sensing material that serves as a bridge between the source (S) and drain (D) electrodes. The interface between the material and the bio‐receptors plays a vital role in the transduction process within FET sensors. To develop an efficient biosensor, considerations such as chemical stability, biocompatibility, and surface functionalization play essential roles in ensuring accurate and reliable detection of target analytes. These factors are important for maintaining the reliability of the biosensor's material during bioreceptor immobilization and target detection processes, thus safeguarding against degradation or adverse chemical reactions. Furthermore, the surface properties of the channel material must facilitate stable attachment of bioreceptor molecules, ensuring specificity and sensitivity in target analyte detection. Additionally, the Debye screening length, which determines the spatial extent of electrostatic interactions in the solution surrounding the biosensor, should be controlled carefully to optimize the sensor's performance for accurate and reliable detection.

A bias voltage is used to change the electrical characteristics of the semiconductor material. The addition of a third electrode, also referred to as the gate, enables the modification of the material's electronic properties, including electrical conductivity. Because of electrostatic gating and/or Schottky barrier modulation, the analytes captured by the sensor induce changes in the material's conductance. This alteration generates a detectable signal which can be recorded and subsequently used to determine the concentration of the analyte.^[^ ^27^ ^]^ FET sensors were initially operated utilizing bulk materials composed of metal oxides (e.g., SnO_2_) and polymeric membranes as the designated sensing channel materials. However, the use of these bulk materials in FET sensors has been constrained by their undesirable electrical properties and insufficient interactions with the target analytes. Furthermore, a large majority of these materials work best only at high temperatures, making them unsuitable for biosensing applications.

The use of 1D and 2D semiconducting nanomaterials as channel materials for FET biosensors has consequently attracted a great deal of attention. The high surface area and nanoscale dimensions of these nanomaterials, which are like the Debye length (D) and hence enable heightened sensitivity, are principally responsible for this increased awareness. For 1D semiconductors, prominent materials used in FET biosensors include conductive polymer nanowires (CPNWs), carbon nanotubes (CNTs), and silicon nanowires (SiNWs). It is crucial to note, though, that there are some restrictions in the ways in which FET biosensors can utilize 1D nanomaterials. The difficulty of obtaining pure conductive or semiconductive CNTs, as opposed to CNTs that are mixed with semiconductive and conductive CNTs, affects their electronic properties. SiNWs also have low carrier mobility and chemical instability, which makes surface passivation necessary.^[^ ^28^ ^]^ Consequently, 2D nanomaterials, including graphene (G), reduced graphene oxide (rGO), transition metal dichalcogenides (TMDCs), phosphorenes, and 2D metal oxides offer enormous potential for FET biosensors.^[^ ^29^, ^30^, ^31^ ^]^ In comparison to their 1D counterparts, these nanomaterials' 2D architectures allow for more reliable and conformal interactions with device electrodes. Thickness and diameter of these 2D nanomaterials can also be precisely adjusted.

Considering all of these discoveries, novel families of 2D materials have been created and are currently expanding quickly. Materials akin to graphene have been developed, encompassing black phosphorus (BP), hexagonal boron nitride (h‐BN), transition metal oxides (LaMnO_3_ and LaVO_3_), transition metal chalcogenides (NbSe_3_ and TaSe_3_), and layered complex oxides.^[^ ^32^, ^33^, ^34^ ^]^ Monolayer and a‐few‐layer transition metal dichalcogenides including MoS_2_, WS_2_, MoTe_2_, MoSe_2_, and WSe_2_^[^ ^35^, ^36^, ^37^ ^]^ have been synthesized. Additional 2D materials like silicene and germanene have also undergone development and are currently under extensive research. A wide variety of materials with various characteristics, ranging from insulators to conductors, are included in the enormous variety of non‐carbon 2D materials. The supplied references contain additional details about the growing collection of 2D elements.^[^ ^38^, ^39^, ^40^ ^]^ As a result, researchers have begun to examine the possible applications of these materials, including FET‐based biosensor systems.

In this comprehensive review, we explore the developing field of electrical devices based on 2D materials for biosensing applications, emphasizing their potential to revolutionize diagnostics and healthcare. With the rising demand for prompt, sensitive, and portable biosensors across various industries, the development of novel materials and sensor designs has become imperative. This review critically investigates the working mechanisms and detection capabilities of biosensing devices utilizing graphene and a wide range of other 2D materials, including transition metal dichalcogenides, and black phosphorus. Incorporating these materials into field‐effect transistor (FET) biosensors offers advantages such as low detection limits (LOD), real‐time monitoring, label‐free diagnosis, and exceptional selectivity. Furthermore, the creation of innovative point‐of‐care diagnostic tools suitable for telemedicine is envisaged with 2D‐based bio‐FETs, alongside a top‐down production approach aligning with existing technology. We also elucidate the diverse applications of these biosensors, from conventional to wearable devices, highlighting the versatility of 2D material‐based FET devices. By interpreting the basic principles governing their operation and showing their advantages over conventional biosensors, we emphasize the potential of 2D materials in advancing biosensing technology. Moreover, this review provides an intensive assessment of the limitations and challenges faced by these devices, besides insights into prospects and advancements. By acknowledging these challenges, we pave the way for future enhancements in biosensor technology, adopting innovation and development in this field. Notably, we present a detailed comparison of FET‐based biosensors, a focal point of our review, compared and tabulated with various other biosensing platforms and their working mechanisms. This comparative analysis offers valuable insights into performance metrics and the suitability of different biosensing platforms for diverse applications. Ultimately, this review aims to stimulate further research and innovation in this appealing area while educating the scientific community about the latest advancements in 2D materials‐based biosensors.

**Figure 2:**
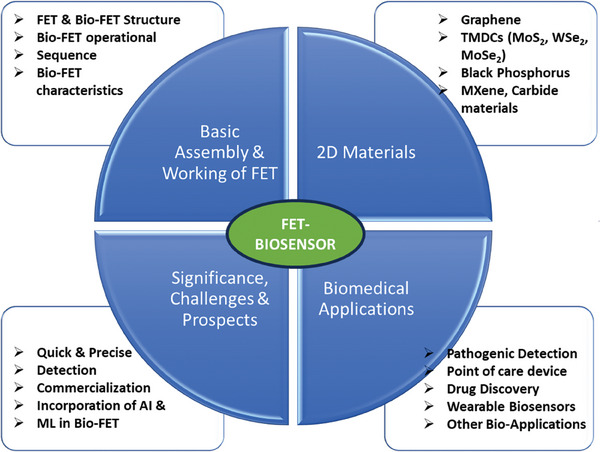
Schematic representation illustrating the basic assembly, working mechanism of 2D materials‐based biosensors, encompassing their significance and potential in biosensing technology.

A scheme illustrating the comprehensive overview, encapsulating the multifaceted aspects explored in this study, is presented in **Figure** **2**.

## 2 Basic Assembly of FET Biosensor Device

Biosensors based on field‐effect transistor (FET) technology have piqued the interest of researchers due to their ability to work in tandem with electronic chips, providing real‐time data while little consuming energy. These biosensors can detect specific compounds without the use of labeling agents and can be made at a reduced cost when created in large quantities.

**Figure 3:**
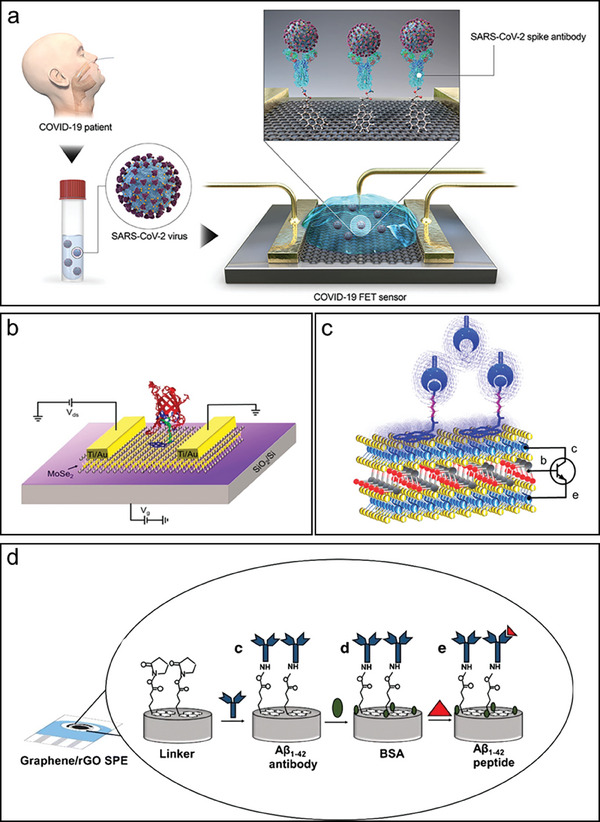
Biosensing device assemblies and structural configurations. a) Schematic for COVID‐19 FET sensor. Graphene is chosen as the sensing material, with the conjugation of SARS‐CoV‐2 spike antibody onto the graphene surface. The graphene‐based FET sensor is characterized with Au/Cr electrodes. Reproduced with permission.^[^ ^44^ ^]^ Copyright 2020, American Chemical Society. b) Illustration presenting the arrangement of the MoSe_2_ field‐effect transistor (FET) atop a p‐doped silicon substrate covered by a 300 nm layer of silicon dioxide. Thin Ti/Au electrodes measuring 5/50 nm were deposited onto the MoSe_2_ sheet. Reproduced with permission.^[^ ^7^ ^]^ Copyright 2023, Elsevier. c) Schematic depiction of the van der Waals (vdW) npn bipolar junction transistor (BJT) biosensing device, featuring supporter molecules composed of pyrene coupled with lysine and biotin, in addition to streptavidin molecules. Reproduced with permission.^[^ ^45^ ^]^ Copyright 2022, Wiley‐VCH. d) The electrochemical system for detecting Aβ1‐42 consisting of a graphene/rGO SPE modified with a linker, an antibody, BSA, and Aβ1‐42 peptide. Reproduced with permission.^[^ ^46^ ^]^ Copyright 2020, Springer Nature.

Typical FETs have a distinguishing component that links the source and drain electrodes and regulates their electrical conductivity via an electric field. Biosensors, on the other hand, use a unique element capable of catching targeted compounds rather than metal electrodes. Such materials carry electric charges that alter the material's conductive capacity, which can be measured by measuring current flow from source to drain.^[^ ^41^, ^42^ ^]^ Silicon dioxide is often used as a separate layer in transistors. However, different substances such as polymers and lipids are used in biosensors. These can be easily integrated into the material of the transistor. Furthermore, a reference electrode is critical in facilitating the process by supplying voltage.^[^ ^43^ ^]^ For instance, a newly created biosensor is presented by Seo, G., et al. for detecting SARS‐CoV‐2 in clinical samples, as depicted in **Figure** **3**a. The biosensor device features a Si/SiO_2_ substrate with a transferred layer of graphene. Graphene is chosen as the sensing material, and the SARS‐CoV‐2 spike antibody is bound to the graphene sheet using 1‐pyrenebutyric acid N‐hydroxysuccinimide ester as an interfacing molecule, serving as a probe linker. Electrodes were created using a metallization process involving chromium (Cr) and gold (Au), resulting device dimensions were 100 × 100 µm^2^ (L × W). Moreover, to reduce interference during electrical measurements, the device was passivated with SU8‐2010.^[^ ^44^ ^]^ Similarly, another biosensor was fabricated by Nisar et al. demonstrating the creation of an innovative detecting system. The n‐type MoSe_2_ material used in this biosensor is placed on top of a Si/SiO_2_ substrate. This biosensor's special quality is its accuracy in identifying the streptavidin protein, which is made possible by the addition of a specially‐made pyrene‐based support molecule. Through *π*–*π* stacking interactions, this support molecule is strategically arranged over the MoSe2 surface. Ti/Au electrodes are placed over the MoSe_2_ sheet to improve the biosensor's performance and give it an extra degree of sophistication. Figure 3b displays a schematic image of the biosensor device, giving a visual description of the creative design and components used in this cutting‐edge technology.^[^ ^7^ ^]^

Similarly, in a separate study, a van der Waals (vdW) bipolar junction transistor (BJT) device was fabricated using GeSe/MoTe_2_ and employed as a biosensor for detecting streptavidin through the utilization of a biotin supporter construct. In order to conduct real‐time measurements in the ambient environment, the van der Waals (vdW) bipolar junction transistor (BJT) devices were configured in the common‐emitter setup as shown in Figure 3c. This diagram showcases a thin GeSe sheet, serving as a base, positioned between the upper MoTe_2_ sheet, acting as the collector, and the lower MoTe_2_ sheet, functioning as the emitter. The functionalization of the vdW BJT devices involved the introduction of a small droplet of dimethylformamide solution containing PLB (1 nm) via a micropipette. This resulted in the stacking of hexagonal pyrene rings over the hexagonal MoTe_2_ sheets through *π*–*π* bonds, as depicted in Figure 3c. Indeed, BJTs offer advantages in terms of signal control and signal amplification compared to FET devices. However, it is essential to note that the fabrication process and its operating mechanism for BJTs can be more complex, often involving the stacking of multiple layers, which may present challenges in manufacturing and scalability

The stacking process facilitated self‐oriented functionalization, leveraging *π*–*π* stacking to prevent non‐specific interactions.^[^ ^45^ ^]^ Moreover, another study has developed a label‐free biosensor that uses a dual‐layer of graphene and electrochemically reduced graphene oxide (rGO) modified with Pyr‐NHS for H31L21 antibody immobilization to detect the plasma‐based Aβ1–42 biomarkers in Alzheimer's disease. Spectral, electrochemical, and morphological methods were used to characterize the alterations. The biosensor demonstrated exceptional selectivity over interfering species and a low limit of detection (2.398 pm) throughout a broad linear range (11 pm to 55 nm) when evaluated using differential pulse voltammetry. Its potential for electrochemical measurement of Aβ1‐42 in bio‐fluidic samples was highlighted by its successful validation in spiking human and mouse plasmas, which correlated with immunohistochemistry (IHC) and magnetic resonance imaging (MRI) results from brain analysis. The fabrication process is visually explained through a schematic representation in Figure 3d.^[^ ^46^ ^]^

**Figure 4:**
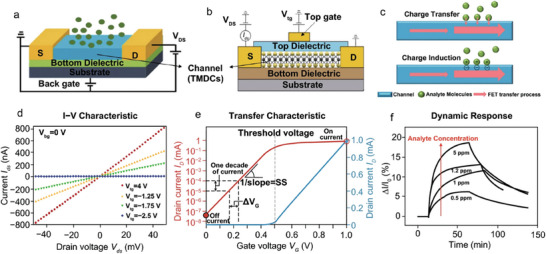
a) Structure of a back gate. Reproduced with permission.^[^ ^48^ ^]^ Copyright 2017, Royal Society of Chemistry. b) a top gate FET with TMDC serving as the channel material. Reproduced with permission.^[^ ^49^ ^]^ Copyright 2011, Nature Nanotechnology. c) the general working principle of FET sensors, involving charge transfer at the interface of the analyte and channel, along with the charge induction effect between analyte molecules and the channel. Reproduced with permission.^[^ ^47^ ^]^ Copyright 2020, Springer. d) The *I*–*V* characteristic (*I*_ds_ vs *V*_ds_ curve) of TMDC FET. Reproduced with permission.^[^ ^49^ ^]^ Copyright 2011, Nature Nanotechnology. e) Transfer characteristic, acquired by monitoring the drain current with forward and backward sweeps of the gate potential. This offers critical parameters of the FET device, including on/off ratio, threshold voltage, and subthreshold slope (ss). Reproduced with permission.^[^ ^50^ ^]^ Copyright 2011, Nature. f) Sensing response is obtained by monitoring the relative change in drain current upon exposure to the target analyte, which varies with the analyte concentration. Reproduced with permission.^[^ ^51^ ^]^ Copyright 2012, Wiley‐VCH.

Connecting smoothly to the technological base of the FET biosensor, the operational sequence starts with the application of the sample, which is usually a clinical specimen containing the target substance, such as SARS‐CoV‐2, starting the FET biosensor's operational sequence. Next, a particular antibody or recognition element designed for the detection of the target material is functionalized onto the FET surface. After functionalization, electrical measurements are made to assess the biosensor's response, which includes looking at current‐voltage (*I*–*V*) curves. The target substance's existence or absence is then ascertained by analyzing the electrical response that was obtained. The system generates an output signal if the biosensor records a positive response, signifying the presence of the target substance. However, if the biosensor is unable to identify the desired material, the procedure may repeat itself or come to an end as a result. The resultant output signal, indicative of the biosensor findings, undergoes additional data processing. To fully comprehend the functioning of the biosensor and the existence or absence of the target material, a more thorough analysis of the electrical measurements and responses obtained is conducted at this phase. Finally, the final phase, which denotes the conclusion of the process, is where the biosensor's operating mechanism culminates. **Figure** **4** shows a schematic diagram of a typical laboratory‐scale study using nanomaterial Bio‐FETs. The stages include device fabrication, testing, electrical characterization, and final biosensing.^[^ ^47^ ^]^ According to their gate‐voltage‐dependent FET behavior, the back gate and top gate of typical FET structures are depicted in Figure 4a,b.^[^ ^48^ ^]^ Figure 4c illustrates the general operation of FET sensors, including charge transfer via the analyte‐channel interface and charge induction between the analyte molecule and the channel. Figure 4d shows the TMDC FET's *I*–*V* characteristic (Ids vs Vds curve).^[^ ^49^ ^]^ Figure 4e illustrates how the gate potential is swept forward and backward to monitor the drain current and acquire the transfer characteristic. It provides important FET device characteristics, such as the subthreshold slope (ss), threshold voltage, and on/off ratio.^[^ ^50^ ^]^ Figure 4f shows how the relative drain current changes in response to the target analyte and how this changes with analyte concentration to determine the sensing response.^[^ ^51^ ^]^

This systematic methodology guarantees the FET biosensor's effective and precise target drug detection, making it a useful instrument for a variety of uses, especially in the context of quick and sensitive diagnostics.^[^ ^52^ ^]^

**Figure 5:**
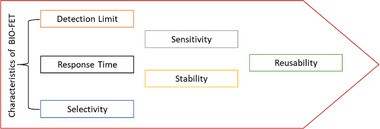
Key characteristics of bio‐FET for a comprehensive understanding of optimal biosensor performance.

Biosensors can be made from a variety of materials, including bulk (3D) and tiny (0D, 1D, 2D). Because of the high surface area to volume ratio, smaller materials are more favored among these possibilities. 2D materials stand out among these options because they feature a thin layer that is suitable for electric conductivity while also being easy to mold and have smooth surfaces. One such example material is graphene, which is well‐known for its remarkable electrical, optical mechanical, and chemical properties.^[^ ^53^ ^]^ Other 2D materials, such as MoS_2_, MoSe_2_, MoTe_2_, SnS_2_, WS_2_ and WSe_2_, have recently gained popularity. These substances offer promise because of a distinguishing feature, that is, when reduced to an ultra‐thin layer, they have energy‐level gaps that distinguish them from graphene in terms of behavior. During fabrication, the n‐type or p‐type electron conductivity can be adjusted. Also, changes to the material's structure can have a substantial impact on its electrical performance.^[^ ^54^ ^]^ The selection and processing of 2D materials in biosensing devices are essential to establishing the device's efficacy. Nanomaterial‐based Bio‐FET parameters, as shown in **Figure** **5**, are essential for assessing the functionality and caliber of the biosensing apparatus. These properties function as crucial markers, facilitating straightforward comparisons between diverse materials and setting standards for assessing performance. Important factors to consider are energy level gaps, electron conductivity, and the capacity to modify electron conductivity during manufacturing. Essentially, these factors serve as measurements for evaluating the biosensing device's overall efficacy in biosensing applications in addition to adding to its variety and adaptability.^[^ ^55^ ^]^

## 3 2D materials‐Based FET Biosensors

**Figure 6:**
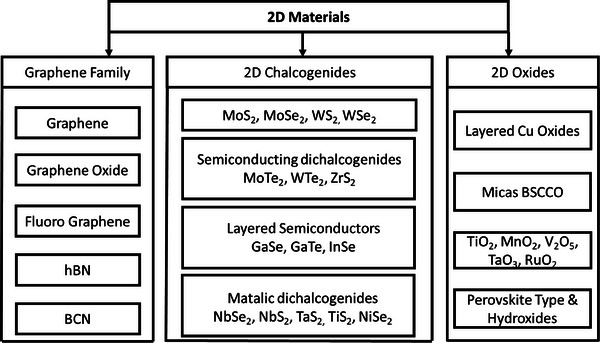
Visualization of the taxonomy of various 2D materials, including graphene.

In 2004, Andre Geim's group at the University of Manchester achieved the remarkable feat of isolating a single sheet of graphite, or graphene. This seminal event acted as a spark for a thorough investigation of 2D materials, resulting in a flood of reviews and articles on their special qualities and wide range of uses. The advent of these materials has brought about a new era of possibilities in micro and nanoscale design, while also revolutionizing sensing technologies, especially in the field of FETs.^[^ ^56^ ^]^ The applications of biosensors and bioelectronics have been further extended by the patterning of 2D materials. The emphasis on patterning these nanomaterials on various substrates has exciting opportunities for wearable medical devices, flexible, portable sensors, and other applications. This paper explores the characteristics of different 2D materials, highlighting their suitability for patterning and their uses in the biosensor field. Moreover, our review will offer a thorough summary of the developments in 2D materials and their possible uses in biosensors. For the reader's ease, the flowchart has been developed regarding the family of layered materials shown in **Figure** **6**.

### 3.1 Graphene

Graphene, a marvel of modern materials science, is a single layer of carbon atoms arranged in a 2D lattice. Despite being incredibly thin (just one atom thick), it exhibits remarkable stability and strength, surpassing even steel in tensile strength. It is a highly versatile 2D material known for its exceptional electrical, mechanical, optical, and chemical properties. Its unique atomic‐thin structure makes it an excellent candidate for various applications, including biosensing. Its planar structure and lack of dangling bonds make it well‐suited as a semiconducting channel in field‐effect transistors (FETs), allowing for precise manipulation of electrical conductivity. This property is harnessed in biosensors, where graphene serves as a platform for capturing target biomolecules, which, in turn, generate a measurable electrical signal. In biosensors, graphene's remarkable surface‐to‐volume ratio and thin atomic layer enable highly sensitive detection of specific biomolecules. Overall, graphene's exceptional properties and ease of functionalization position it as a pivotal material in the field of biosensing, holding significant potential for a broad spectrum of applications in healthcare and biotechnology.^[^ ^57^ ^]^

**Figure 7:**
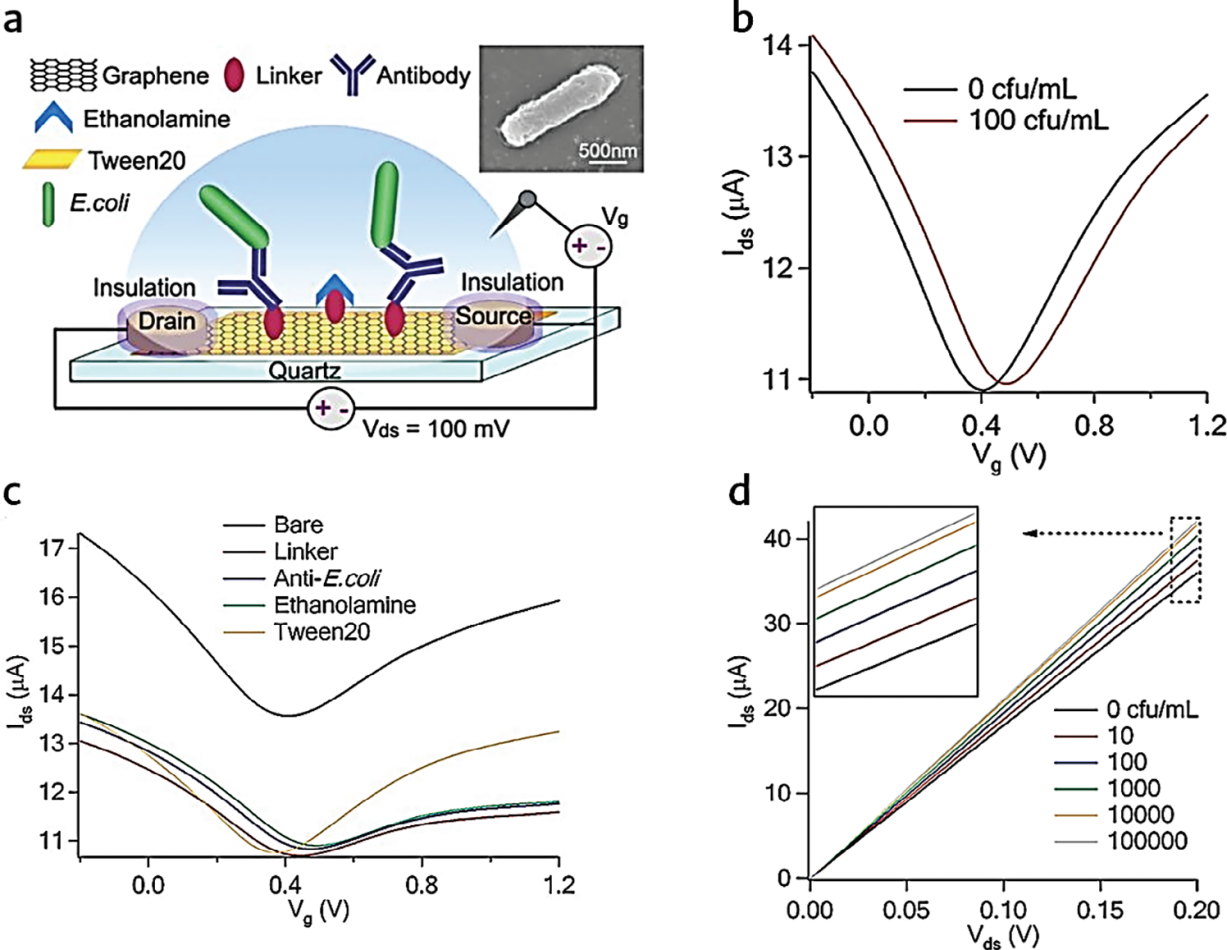
a) Illustration of a FET sensor employing graphene for the detection of *E. coli* bacteria. b) FET *I*_ds_‐*V*_g_ curve before and after incubation with *E. coli* bacteria at a concentration of 100 CFU mL^−1^. c) *I*_ds_‐*V*_g_ curves for bare graphene, linkers, anti‐*E. coli*, Ethanolamine, and Tween20. d) *I*_ds_‐*V*_ds_ characteristics observed at varying concentrations of *E. coli*. Reproduced with permission.^[^ ^59^ ^]^ Copyright 2011, Royal Society of Chemistry.

An FET uses an electric field to control the shape and conductivity of a pathway for electric charge in a material that conducts electricity partially. In this case, FETs made with reduced graphene have been widely used for detecting DNA combinations, negatively charged bacteria, and a specific type of protein called IgE on graphene surfaces. They have also been effective in identifying proteins in a liquid solution. Scientists discovered that a modified version of graphene‐FET, using a molecule called aptamer, successfully detected the IgE protein.^[^ ^58^ ^]^ Moreover, in another study, Yinxi et al. investigated the potential applications of graphene in biosensing. A graphene‐based biosensor for the very sensitive and precise detection of *E. coli* bacteria is fabricated. For the fabrication, a huge graphene film was produced and functionalized with anti‐*E. coli* antibodies and a passivation layer using chemical vapor deposition. The graphene‐based biosensor showed no reaction to another bacterial strain. However, a significant increase in conductance is observed when exposed to low quantities of *E. coli* (10 cfu mL^−1^). Real‐time monitoring of glucose‐induced metabolic activity inbound *E. coli* bacteria was also accomplished by the gadget. It is a simple, quick, and label‐free nanoelectronics biosensor that shows potential as a high‐throughput platform for the detection of various pathogenic bacterium and its functionality.^[^ ^59^ ^]^ This extensive use of graphene and MoS_2_‐based FETs in biosensors for detecting *E. coli* bacteria is shown in **Figure** **7**a, by Yinxi et al. To achieve specificity, chemically vapor‐deposited (CVD) graphene is grown, followed by functionalization with linkers and anti‐*E. coli* antibodies. Figure 7b depicts the fascinating phenomena in which *E. coli* causes holes in the graphene sheet, increasing conductivity on the left side of the Dirac point. Figure 7c displays the drain current‐gate voltage (I_ds_‐V_LG_) transfer curve, highlighting the processes of functionalization with linkers, anti‐*E. coli*, ethanolamine, and tween 20. Figure 7d depicts the drain current‐drain voltage (*I*_ds_–*V*_ds_) performance at various *E. coli* bacteria concentrations, revealing the sensor's detection limit of 10 CFU mL^−1^.

**Figure 8:**
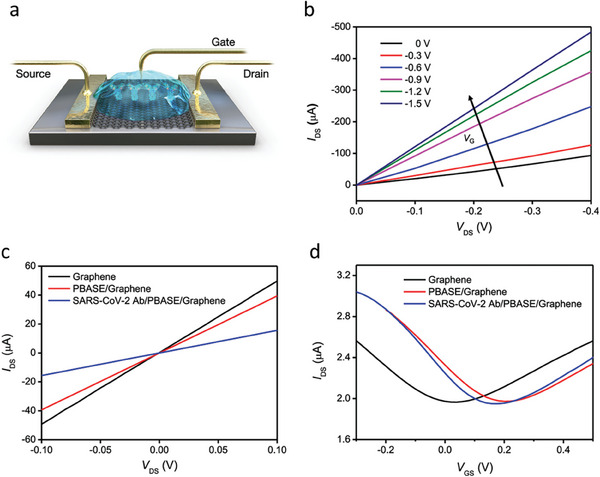
Electrical analysis of graphene in different states: pristine, PBASE‐modified, and graphene with immobilized SARS‐CoV‐2 spike antibodies. a) schematic representation of the configuration using an aqueous‐solution gated FET, also known as the COVID‐19 FET sensor, employing antibody‐conjugated graphene. b) output curves (IDS–VDS) of the antibody‐conjugated FET under various gating voltages, ranging from 0 to −1.5 V in increments of −0.3 V. Notably, IDS increases as the negative gating voltage (VGS) increases. c) Current–voltage (*I*–*V*) characteristics of the graphene‐based device at different stages of functionalization for antibody modification. d) Measurement of transfer curves for the COVID‐19 FET sensor during antibody conjugation steps, with a constant VDS of 0.01 V. Reproduced with permission.^[^ ^44^ ^]^ Copyright 2020, American Chemical Society.

In another work, researchers developed a novel gadget that uses a FET to identify SARS‐CoV‐2 in patient samples using graphene. An antibody that detects the virus's spike protein was used to coat the FET. Several samples from COVID‐19 patients were used to test the device, and the results showed that it could successfully detect the virus at extremely low amounts.^[^ ^44^ ^]^ In this work, electrical measurements were used to assess the SARS‐CoV‐2 spike antibody's presence on a graphene surface, as shown in **Figure** **8**a. The graphene device's–voltage (*I*–*V*) curves at various biasing voltages are presented in Figure 8b. Following PBASE functionalization and antibody immobilization, the slopes (dI/dV) dropped, showing that the SARS‐CoV‐2 spike antibody had been successfully introduced. Moreover, an FET sensor with aqueous solution gating was developed to assess the electrical signal transduction capability of the COVID‐19 FET sensor. Figure 8c illustrates the detection of SARS‐CoV‐2 through variations in channel surface potential and the subsequent electrical response. Following each modification operation, the graphene‐based FET's transfer curves were measured (Figure 8d). Following PBASE functionalization, a favorable shift was observed due to the p‐doping impact of the pyrene group. However, the transfer curve exhibited a negative shift after antibody immobilization, suggesting the n‐doping effect caused by the positive charge of the antibody on graphene. The output curves of the COVID‐19 FET sensor as a function of gate voltage (VG) are shown in Figure 8b, which illustrates how a p‐type semiconductor is expected to behave. The reliability of the COVID‐19 FET sensor in producing an electrical signal to detect target analytes like the SARS‐CoV‐2 antigen protein, cultured SARS‐CoV‐2 virus, or the virus in clinical samples is indicated by the consistent ohmic contact demonstrated in the linear *I*–*V* curves.

The extraordinary qualities of graphene, such as its unmatched mechanical strength, flexibility, and conductivity, highlight its revolutionary significance as a material with far‐reaching effects in a wide range of scientific and technological fields. The absence of a bandgap limits graphene's direct application in certain semiconductor scenarios despite the graphene biosensor's high sensitivity, simplicity of production, fast reaction time, and label‐free detection. This issue has prompted researchers to focus on single‐layer semiconducting materials with appropriate bandgaps. Transition metal dichalcogenides monolayers have attracted much attention because of their unique features in the optical, electrical, and physical domains. This study underscores the potential influence of 2D materials on biosensing.

### 3.2 Transition Metal Dichalcogenides

Transition metal dichalcogenides (TMDCs) are the family of layered materials represented by the generic formula MX_2_, where X is an atom of a chalcogen (S, Se, or Te) and M is a transition metal from groups IV, V, or VI (e.g., W,Mo, Cr, Ti, Zr, Hf; V, Nb or Ta). Chalcogen, transition metal, and chalcogen make up the three planes that make up the basic structure of TMDCs. Interestingly, TMDCs can exist in several coordination states, in which six chalcogen atoms are grouped around each transition metal atom in an octahedral or triangular prism structure. Because of their distinct structure and unique composition, TMDCs are a unique class of materials with potential uses in a variety of industries.^[^ ^60^, ^61^ ^]^

The electrical characteristics of TMDCs range from metallic (NbS_2_, VSe_2_)^[^ ^62^, ^63^ ^]^to semi‐metallic (WTe2, TiSe2),^[^ ^64^, ^65^ ^]^ semiconducting (MoS_2_, MoSe_2_, WS, WSe_2_),^[^ ^66^, ^67^ ^]^ and insulating (HfS2).^[^ ^68^ ^]^ MoS_2_ is the first well‐known semiconducting TMDC that caught the attention of researchers for field‐effect transistor applications because of its high on/off current ratio.^[^ ^69^ ^]^ Except for GaSe and ReS_2_, most TMDCs parade an indirect bandgap (bulk form) that changes to a larger direct bandgap (monolayer form). Remarkably, TMDCs with no dangling bonds, such as MoS_2_ and WSe_2_, create perfect Schottky junctions that avert the transfer of charges at the interface through bulk metals. Grain boundaries, substrate choice, and metal contacts are some of the variables that affect mobility in TMDCs.^[^ ^70^, ^71^ ^]^ Therefore, semiconducting TMDCs are the best options for FET applications. Among these FET based biosensors, MoTe_2_, MoS_2_, MoSe_2_, WS_2_, and WSe_2_ are semiconducting TMDCs that are highly favored as materials. Significant advantages include minimal leakage current, low power consumption, and a high current on/off ratio. These characteristics make semiconducting TMDC‐based FET biosensors more sensitive and attractive options for a range of sensing applications. Here, our study now presents research that utilizes TMDC materials in the fabrication of biosensors.

**Figure 9:**
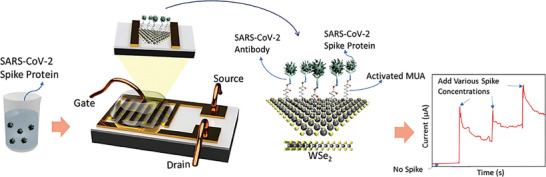
Field‐effect transistor (FET) based COVID sensor fabricated using a WSe_2_ monolayer. The sensing material was monolayer WSe_2_ crystals with interdigitated electrodes manufactured on them. Using 11‐mercaptoundecanoic acid (MUA) to immobilize the SARS‐CoV‐2 antibody on the WSe_2_ crystals, n‐hydroxysuccinimide (NHS) and carbodiimide hydrochloride (EDC) solution were used to activate the antibody. The SARS‐CoV‐2 spike protein might be detected by the 2D‐FET device by observing its impact on the electrical transport characteristics. Reproduced with permission.^[^ ^72^ ^]^ Copyright 2021, American Chemical Society.

Fathi‐Hafshejani, P., et al. reported the quick and sensitive detection of SARS‐CoV‐2 using a field‐effect transistor,^[^ ^72^ ^]^ constructed using the semiconducting transition metal dichalcogenide WSe_2_. **Figure** **9** shows the schematic diagram of the created monolayer WSe_2_‐based FET COVID sensors. The sensing material utilized in this study was monolayer WSe_2_ crystals, on which interdigitated electrodes were fabricated. The SARS‐CoV‐2 antibody was immobilized onto the WSe_2_ crystals using 11‐mercaptoundecanoic acid (MUA), which was activated using a solution of n‐hydroxysuccinimide (NHS) and carbodiimide hydrochloride (EDC). Moreover, when a monoclonal antibody specific to the SARS‐CoV‐2 spike protein is used to functionalize WSe_2_ monolayers, it achieves an impressive detection limit of 25 fg µL^−1^ in 0.01X phosphate‐buffered saline (PBS). To describe and clarify the functioning of the device, a thorough examination combining theoretical and experimental methods, such as atomic force microscopy, Raman and photoluminescence spectroscopies, and an evaluation of electronic transport properties was carried out.

**Figure 10:**
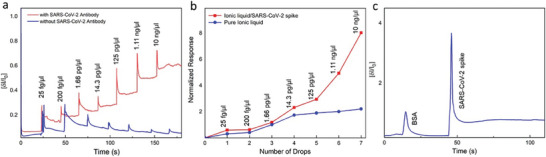
Real‐time detection of the SARS‐CoV‐2 antigen spike protein using a WSe_2_ COVID‐19 2D‐FET. a) The sensor's real‐time response is displayed for different SARS‐CoV‐2 antigen spike protein concentrations under particular voltage circumstances (VDS = 1 V, VGS = −0.5 V), both with and without the presence of antibodies (blue curve). b) Response plots of an ionic liquid (0.01X PBS) with and without the SARS‐CoV‐2 spike protein are compared. c) Selective response of the COVID‐19 FET sensor is demonstrated with respect to the SARS‐CoV‐2 antigen protein as well as the target BSA. Reproduced with permission.^[^ ^72^ ^]^ Copyright 2021, American Chemical Society.

Furthermore, based on the associated alterations in the electrical transport characteristics, the study examined the COVID‐19 2D‐FET sensors' real‐time response and assessed how well they performed with varying spike protein concentrations. When functionalized with SARS‐CoV‐2 antibodies, **Figure** **10**a shows the sequential changes in device output with different spike concentrations, showing effective interaction. The antibody‐free gadget, on the other hand, displayed very little reaction (blue line). The sharp contrast in the device response with increasing spike concentrations is seen in Figure 10b, which shows additional testing to differentiate spike detection from an increase in ionic volume. The selectivity of the biosensor is notably illustrated in Figure 10c, where responses to 10 ng µL^−1^ SARS‐CoV‐2 spike antigen protein and 10 ng µL^−1^ bovine serum albumin (BSA) proteins are compared. The COVID‐19 FET sensor based on WSe_2_ showed a negligible reaction to BSA proteins, but it responded specifically to the SARS‐CoV‐2 spike antigen protein. These results underscore the potential of WSe_2_‐based FET devices as highly sensitive and selective biosensors for SARS‐CoV‐2 detection.^[^ ^72^ ^]^

**Figure 11:**
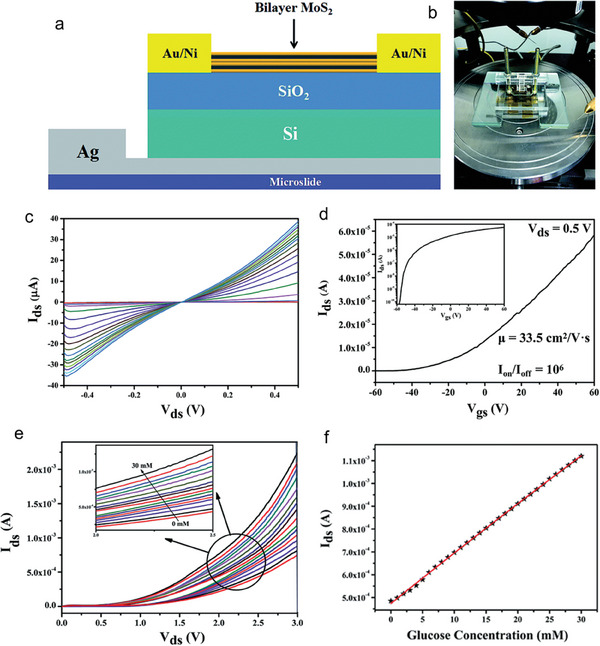
Biosensor device utilizing MoS_2_‐based FET. a) Schematic representation of the bilayer MoS_2_‐based FET. b) Visual depiction of the sample cell, with the glucose solution injected into the rectangular hole. c) Output characteristic curve showing the drain voltage (Vds) versus drain current (Ids). In 5 V increments, the back gate voltage (Vgs) ranges from −40 to 40 V. d) Transfer characteristic curve with a constant drain voltage (Vds) of 0.5 V, displaying the drain current (Ids) as a function of back gate voltage (Vgs). The transfer characteristic curve's logarithmic representation (Ids–Vgs) is shown in the inset. e) The graph shows the drain current variation as a function of Vds for an n‐type MoS_2_ FET‐based glucose sensor at various glucose solution concentrations. f) The reaction of the MoS_2_ FET to a glucose solution, shows Ids (drain current) as a function of glucose concentration, which ranges from 0 to 30 mm. Reproduced with permission.^[^ ^77^ ^]^ Copyright 2018, Royal Society of Chemistry.

Recently, several biosensor devices have been reported to detect glucose by utilizing the 2D materials based biosensor devices.^[^ ^73^, ^74^, ^75^, ^76^ ^]^ Shan, J., et al. reported on MoS_2_‐FET‐based glucose biosensor.^[^ ^77^ ^]^ This research investigates the application of FETs that are based on molybdenum disulfide (MoS_2_) as biosensors for the detection of glucose solutions. MoS_2_ nano‐materials, renowned for their exceptional properties, particularly as an optimal channel material, have garnered significant attention. The study entails the creation of a FET, constructed using bilayer MoS_2_ and evaluating its effectiveness in detecting extremely low concentrations of glucose solutions. The MoS_2_ FET's structure is illustrated in **Figure** **11**a. Alongside the FET, a sample cell was essential. Constructed from plexiglass, the sample cell was designed in three parts and assembled by screwing it together with the MoS_2_ FET. Figure 11b presents the physical diagram of the sample cell. The electrical characteristics of the MoS_2_ FET significantly influence the biosensor's sensitivity. Figure 11c,d presents the assessment of the electrical properties of the MoS_2_ nanosheet‐based FET in the absence of a glucose solution. In Figure 11c, the output characteristic (*I*_ds_–*V*_ds_) curve indicates that Ids increases with the rise in *V*ds. Particularly in the low voltage region of Vds, a linear increase in *I*ds with *V*ds is observed, signifying the formation of good ohmic contact between the electrodes and MoS_2_ layers. This is advantageous for electron injection. The device's effective control by Vgs is demonstrated in the output characteristic curve with different *V*_gs_ in Figure 11c.

Moreover, key electrical parameters of the MoS_2_ FET are derived from the transfer characteristic curve (*I*_ds_–*V*_gs_) and the logarithmic image of *I*_ds_–*V*_gs_, as shown in Figure 11d. Calculations reveal a field‐effect mobility (µ) of 33.5 cm^2^ V⁻¹ s⁻¹, a transconductance (gm) of 1.7, and a high switching current ratio (Ion/Ioff) reaching 106. The biosensor demonstrates remarkable sensitivity, with a calculated value of 260.75 mA mm ^−1^ and a proven measurable detection limit of 300 nm. Moreover, the article illustrates the successful identification of unknown glucose solution concentrations based on the relationship between source‐drain current (I_ds_) and glucose concentration (nm). Furthermore, the biosensor exhibits advantageous characteristics including a prompt response time (<1 s), excellent solidity, an extensive linear detection limit ranging 300 nm to 30 mm, and the capability to minutely detect the glucose concentration in solution form. These distinctive attributes position the bilayer MoS_2_‐FET as a promising contender for the forthcoming generation of biosensors. MoS_2_ FET sensor for glucose sensing is illustrated in Figure 11e,f. At constant Vg and Vsd, Figure 11e,f illustrates the direct proportionality of current (Ids) to glucose concentration with a limit of detection (LOD) of 300 nm as well as a sensitivity of 260.75 mA mm ^−1^. This association is caused by electrons from the oxidation of glucose doping MoS_2_ to an n‐doping state. To accurately determine unknown glucose concentrations, the study additionally makes use of a calibration curve, which plots Isd versus glucose concentration.^[^ ^77^ ^]^

**Figure 12:**
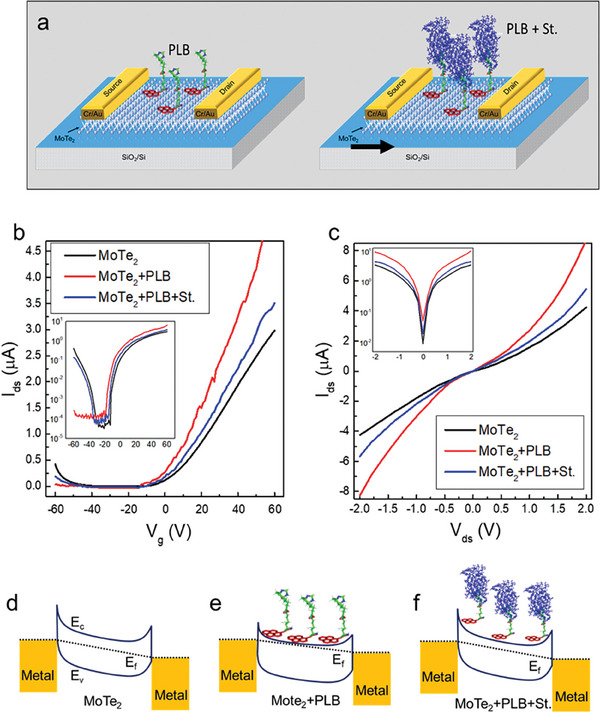
a) Schematic image to show the supporter construct (nominated as PLB) that is decorated on the MoTe_2_ field‐effect transistor surface. It also shows how the streptavidin interacts with the supporter moiety in a specific way called *π*−*π* stacking b) The transfer curves for the MoTe_2_ at a fixed Vds = 1 V (pristine‐black), after applying supporter moiety (functionalized‐red), and after introducing streptavidin target (protein detection‐blue), has been presented. The inset displays the transfer curves on a logarithmic scale. c) The pristine MoTe_2_ (shown in black), after functionalization (shown in red), and after target capturing (shown in blue) Ids‐Vds curves are displayed at a constant gate voltage (Vg) of +60 V. The Ids–*V*ds curves in logarithmic scale are shown in the inset of the figure. d−f) Energy band diagrams depict the shift in Fermi‐level and variations in barrier height for (d) pristine MoTe_2_ FET, (e) post‐stacking of PLB moiety, and (f) subsequent to capturing target protein. Reproduced with permission.^[^ ^78^ ^]^ Copyright 2023, American Chemical Society.

Nisar, S., et al. developed an FET‐based biosensing device that can quickly detect the protein streptavidin (St.).^[^ ^78^ ^]^ A few layers of molybdenum ditelluride (MoTe_2_) on a p‐doped Si/SiO_2_ substrate are used to produce the MoTe_2_‐FET‐based biosensor. MoTe_2_‐based FET for biosensing applications was evaluated by employing a probe station, atomic force microscopy (AFM), and Raman spectroscopy, among other characterization techniques. Pyrene‐encumbered lysine–botin (PLB) was used in a self‐engineered supporting construct to enable the detection of streptavidin at various concentrations. **Figure** **12**a is the schematic representation of FET‐Biosensors utilizing MoTe_2_ materials, highlighting the substrate, drain‐source, PLB supporter construct, and target analyte. Figure 12b shows the transfer curves for the pristine MoTe_2_, presented in black, followed by its functionalizing using the supporter construct (PLB), presented in red, and finally after adding the target biomolecule streptavidin, presented in blue. Each curve's logarithmic plot is indicated by the inset. It can be observed that the introduction of the PLB molecule results in the drastic increase of device current.

The increase in current has been attributed to the relatively high electron affinity of the supporter construct (PLB), which shares electrons with the channel material containing MoTe_2_ upon functionalization. Lastly, a notable drop in current was noticed when a solution‐carrying target biomolecule (1 pm streptavidin) was drop‐casted onto the functionalized MoTe_2_ surface. This decrease indicates that the PLB supporter construct has been successful in capturing the streptavidin molecules, which was made possible by their strong noncovalent bonding contacts. Furthermore, the Ids−Vds curves are depicted in Figure 12c at a constant gate voltage of Vg = +60 V for pristine MoTe_2_ (shown in black), post‐functionalization with the PLB supporter construct (shown in red), and introduction of streptavidin as a biomolecule (shown in blue). The Ids‐Vds curves exhibit a nonlinear tendency even after the MoTe_2_‐FET was introduced with PLB and streptavidin. Furthermore, Figure 12d–f describes the mechanism of charge transfer by utilizing energy band diagrams. In the case of pristine MoTe_2_ sheets, the Fermi level (E_f_) is located close to the conduction band (E_c_), slightly distanced from the valence band (Ev). This establishes a substantial barrier height, which has been attributed to the Schottky barrier contribution originating from the metal electrodes, as illustrated in Figure 12d. With the introduction of PLB molecules into the MoTe_2_ sheet, the Fermi level experiences an upward shift due to electron sharing, as demonstrated in Figure 12e. The observed upward shift is a consequence of extensive electron sharing between the negative charge of the PLB supporter construct and the MoTe_2_ FET surface. This interaction leads to a reduced barrier height and an amplified device current. Subsequently, upon exposure to a solution containing the target protein (streptavidin), the PLB molecules transfer their charge to the streptavidin, resulting in a significant reduction in device current. This reduction signifies a moderate increase in the barrier height between MoTe_2_ and the metal electrodes, accompanied by a downward shift in the Fermi level, as illustrated in Figure 12f.

**Figure 13:**
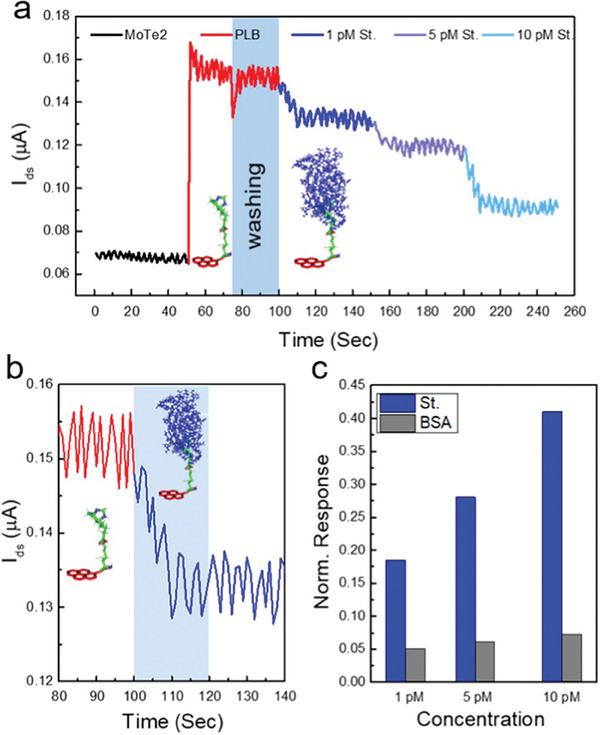
MoTe_2_ FET measurement in real‐time for the target protein (streptavidin) detection. a) An unchanging Vds of 1 V was placed between the source‐drain while keeping zero gate voltage and the current was monitored and recorded continuously. The device response after functionalization is presented by the red line, while the black line depicts the clean MoTe_2_ FET's current (in the pristine state). The blue lines in various hues show how the device current reduces as streptavidin is added at increasing concentrations. b) MoTe_2_ FET's temporal reaction to the identification of 1 pm streptavidin. The response time, or 19 s, is shown in the blue‐shaded zone. c) To demonstrate the selectivity of the device, the bovine serum albumin (BSA), a non‐targeted protein with a molecular weight like streptavidin, was used. As evidenced by the gray bar graphs, the MoTe_2_ FET remains unresponsive to the BSA protein, even when its concentration is increased. Reproduced with permission.^[^ ^78^ ^]^ Copyright 2023, American Chemical Society.

Additionally, the current of the MoTe_2_‐FET was measured with streptavidin at different concentrations, and the accompanying change in current was also measured. A rapid device response and a significant shift in current were seen as the target protein concentration increased from 1–10 pm, as presented in **Figure** **13**a. A temporal response for MoTe_2_‐FET was computed while detecting minute solution concentration (1 pm streptavidin). The ultimate time response of MoTe_2_‐FET was recorded at ≈19 s and can be nominated as the LOD, as Figure 13b illustrates. Furthermore, selectivity of MoTe_2_‐FET against bovine serum albumin (BSA), which has a molecular weight comparable to streptavidin, was also investigated. Comparing the MoTe_2_‐FET to the streptavidin, as shown in Figure 13c, there was no discernible reaction to a standard concentration of BSA available. This work investigates how the MoTe_2_‐based biosensor responds dynamically to different streptavidin concentrations, from 10 to 1 pm.^[^ ^78^ ^]^

**Figure 14:**
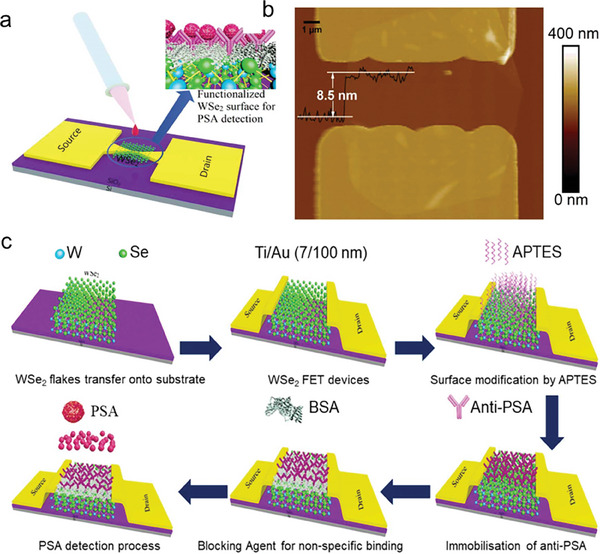
PSA sensor WSe_2_ FET. a) Schematic of the WSe_2_ FET sensor used in PSA detection. b) Image from atomic force microscopy (AFM) showing the WSe_2_ semiconducting channel's surface. c) Flowchart detailing the process of surface functionalization and fabrication. Reproduced with permission.^[^ ^13^ ^]^ Copyright 2021, IOP Publishing.

Another research study describes the creation of a highly sensitive FET‐biosensor by decorating tungsten diselenide (WSe_2_) that can be used to detect early‐stage prostate cancer without the need for a label. To achieve precise binding between the anti‐PSA and PSA, a multi‐step approach including the attachment of the antibody (monoclonal) relating to prostate‐specific antigen and subsequent treatment with bovine serum albumin is used to modify the FET channel.^[^ ^13^ ^]^ **Figure** **14**a–c illustrates the WSe_2_ FET PSA sensor schematics image, AFM, and stepwise fabrication process respectively. The biosensor reports the lowest concentration of FET, a stunning LOD of 10 fg mL^−1^ PSA. The sub‐threshold swing is 235 mV dec^−1^ at this detection limit, and the drain current (Id) rises by 70% above a pristine device's value. Such parameters as threshold voltage (Vth) and drain current (Ids) of the FET sensor show a linear variation while the range of linear detection is from 10 fg mL^−1^  to 1 ng mL^−1^ for PSA. The demonstrated ultra‐sensitive and prompt WSe_2_ FET biosensors have a lot of potential for use in point‐of‐care diagnostic procedures.^[^ ^13^ ^]^

Ma, M., et al. fabricated an inorganic nano MoS_2_‐FET‐based biosensor for identifying the precise relationship between sugar and lectin. This interaction is important for diagnosing and developing new drugs, as well as for understanding a variety of human diseases. The biosensor is produced by a straightforward assembly procedure that includes the immobilization of probe molecules (D‐Mannose) on the FET by Schiff base reaction, the functionalization of gold nanoparticles on the MoS_2_ surface, and the self‐assembly of β‐mercaptoethylamine on gold via Au–S interactions. Analyses using a scanning electron microscope and x‐ray photoelectron spectroscopy verify the FET sensor's interaction with Concanavalin A (ConA) and the assembly process. With a detection limit of less than 105 nm, the manufactured FET biosensors demonstrate accurate detection of the target ConA.^[^ ^79^ ^]^

### 3.3 Black Phosphorus

**Figure 15:**
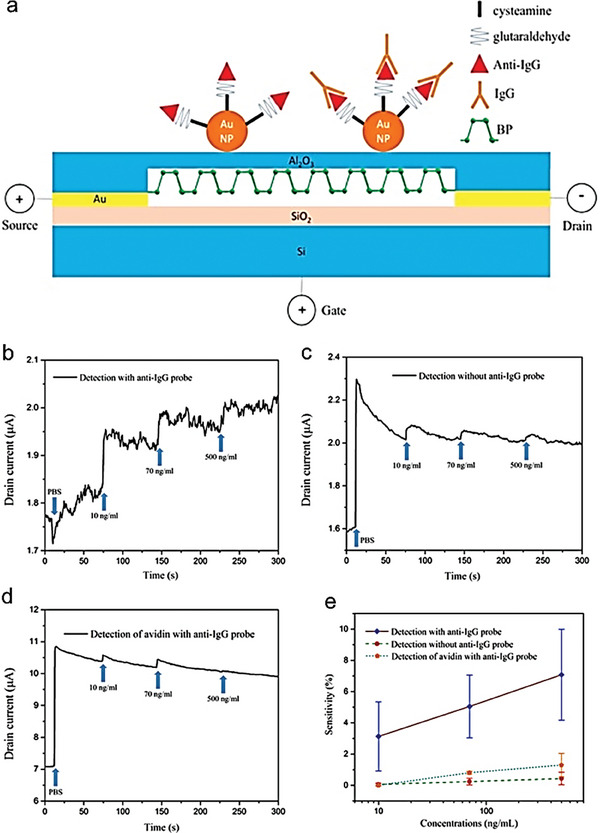
a) Schematic of the BP‐FET sensor. The source‐drain electrodes are bridged by BP nanosheets that are physically exfoliated and deposited onto the device. Chemically conjugated antibody probes with Au NPs are used. The BP nanosheet surface is passivated using the Al_2_O_3_ dielectric layer to stop oxidation. b) The sensor's dynamic response to changing IgG concentrations. c) Control experiment evaluating the binding capability of non‐specific antibodies to the sensor surface and without antibody probes. d) The control experiment was to check the binding of a non‐specific protein (avidin). The source‐drain voltage (Vds) was fixed at 0.01 V throughout the experimental investigation. e) Plotting sensitivity against the concentration of the target protein. The sensitivity is achieved by binding of specific IgG molecules on the channel of the device, as opposed to devices exposed to non‐specific proteins or those lacking probes. Reproduced with permission.^[^ ^81^ ^]^ Copyright 2017, American Chemical Society.

Previous research focuses on phosphorene biosensors and examines the uses of black phosphorus (BP) and phosphene in biomedicine and biosensing.^[^ ^80^ ^]^ To detect human immunoglobulin G (HIgG), Chen et al.^[^ ^81^ ^]^ created a FET biosensor employing a few‐layer BP nanosheets passivated with an Al_2_O_3_ layer. Anti‐HIgG biorecognition molecules were immobilized using gold nanoparticles. The BP‐FET device, in which antibody probes are immobilized on the BP nanosheet to facilitate binding with antigens, is illustrated schematically in **Figure** **15**a. On the BP sensor, scanning electron microscopy (SEM) was used to examine the device's structure as it was built. Moreover, the electrical parameters of the gadget were characterized, and it demonstrated p‐type behavior. A quick reaction was seen in tests using various HIgG antigen concentrations, with a LOD of up to 10 ng mL^−1^ for a target molecule. Moreover, it provides important insights into the efficacy and specificity of the BP FET biosensor in detecting target proteins by elucidating its dynamic response, sensitivity, and selectivity characteristics under diverse experimental settings. The dynamic response of the device containing BP antibody probes is illustrated in Figure 15b. When the increasing concentration of IgG from 10 to 500 ng ml^−1^, the KPI‐sensitivity, which is measured as ΔG/G0 or ΔI/I0, increases from 5% to 10%. In this presentation, ΔG/I is the change in electrical conductivity or Ids, and G0/I0 is the base conductivity or current before antigen injection. Interestingly, the sensor generates a distinguishable prompt response against the target protein, highlighting its robust reaction time. Likewise, Figure 15c shows a control experiment with a BP device that does not have any anti‐IgG probes. Although there is a modest increase in current responses to IgG in this arrangement, these responses are unstable and return to their previous values in 20–25 s. Another control experiment in Figure 15d uses avidin (non‐specific protein), to investigate selectivity of the BP device. The response of the BP biosensor with anti‐IgG probes is comparable to that of the device without probes, suggesting that proteins bind to the BP surface non‐specifically and provide fleeting signals. Figure 15e offers a comparison of sensor sensitivity under various conditions. Comparing anti‐IgG immobilized devices to control groups, the former exhibits noticeably higher sensitivities. Probe‐free devices show very little sensitivity, even when the IgG concentration rises, ranging 10–500 ng ml^−1^. In a similar vein, instruments with non‐specific proteins (avidin) also show low sensitivity. This validates the BP‐based FET biosensor's exceptional selectivity for the target protein (IgG).

Similar working principles are shared by the BP‐based FET biosensor and previously disclosed semiconducting 2D materials like MoS_2_ and reduced graphene oxide (rGO). For instance, according to Mao et al., a vertically oriented G‐FET sensor can detect the IgG down to a limit of 2 ng ml^−1^.^[^ ^82^ ^]^ Moreover, a few‐layer black phosphorus‐based biosensor was effectively built by Kim et al.^[^ ^83^ ^]^ with the specific goal of detecting alpha‐fetoprotein (AFP), which is known to be a highly dependable tumor marker for hepatocellular carcinoma diagnosis. The immobilization of AFP antibodies was achieved using a poly‐L‐lysine linker in the surface functionalization of the biosensor. The biosensor showed the capacity to identify different AFP antigen concentrations, ranging from 1 ppm to 0.1 ppb, via the selective binding of AFP antigens and antibodies. The results depicted a noteworthy relationship (linear) between the AFP levels and corresponding electrical current, highlighting the excellent sensitivity of the biosensor in detecting AFP. By making use of the fact that phosphorus is the second most abundant mineral in the human body, making up ≈1% of body weight, and the fact that the products of black phosphorus's biodegradation are biocompatible, Song et al. creatively created a BP‐FET. The BP‐FET exhibited remarkable endurance in bodily fluid, with elevated mobility and an ON/OFF current ratio for a time of ≈36 h, before it completely disintegrated. With its special qualities, this transient biocompatible biosensor possesses the potential to pave new opportunities for momentary biosensing applications.^[^ ^84^ ^]^

### 3.4 MXenes

MXenes are a family of 2D materials composed of transition metal carbides, nitrides, or carbonitrides. They are derived from layered ternary carbide and nitride precursors, known as MAX phases, via selective etching of the A‐group element (usually aluminum). MXenes exhibit a distinctive combination of properties, including high electrical conductivity, mechanical flexibility, and tunable surface chemistry. These characteristics make MXenes favorable candidates for a wide range of applications, including energy storage, catalysis, and sensing. In biosensor applications, MXenes have demonstrated agreat potential due to their large surface area, chemical stability, and ability to facilitate electron transfer reactions.^[^ ^85^ ^]^ Their surface chemistry can be modified to enhance biocompatibility and selectivity for specific biomolecules, making them more suitable candidate for detecting various analytes with high sensitivity and specificity. Furthermore, MXenes can be easily integrated into sensor platforms, suggesting opportunities for the development of next‐generation biosensing devices with improved performance and functionality. Continued research into MXene‐based biosensors holds promise for advancing healthcare diagnostics, environmental monitoring, and other diagnostic applications.

### 3.5 Nitride and Carbonitride‐Based Materials

The nitride‐based materials can be used for biosensing applications using FETs. Specifically, silicon‐based materials have traditionally dominated the field of biosensors, and nitride‐based materials, such as silicon nitride (Si3N4) and boron nitride (BN), have gained attention due to their unique properties that make them suitable for biosensing. In addition to the above, hexagonal boron nitride (h‐BN) offers several advantageous properties that make it attractive for biosensing applications. The h‐BN is well‐tolerated by biological systems and does not induce significant immune responses or toxicity. This property makes it feasible to interact with biological samples without causing adverse effects. Moreover, it is chemically inert, which means it does not readily react with most chemicals or biomolecules. This inertness reduces the likelihood of interference or fouling during biosensing experiments, leading to more reliable and accurate results. In addition to the above, it has a smooth atomic surface and relatively high mechanical strength which makes it a suitable candidate for biosensing applications where the sensor may be subjected to mechanical stresses or repeated use.^[^ ^86^, ^87^, ^88^, ^89^ ^]^ The silicon nitride (Si3N4) is also known for its excellent properties to be used in microelectronics. The material's high biocompatibility and stability make it suitable as a substrate for immobilizing biomolecules such as antibodies, enzymes, or DNA probes. The Si3N4‐based biosensors can detect biomolecular interactions through various transduction mechanisms, including electrical, optical, and mechanical sensing.^[^ ^90^, ^91^, ^92^, ^93^ ^]^ In addition to the above, the boron carbonitride (BCN) materials, which are composed of boron, carbon, and nitrogen, have garnered interest for their potential applications in various fields, including biosensing. While research in this area is still in its early stages, BCN materials hold promise for biosensing due to its tunable properties, chemical stability, high electrical conductivity, mechanical strength, biocompatibility, and enhanced surface area available for binding.^[^ ^94^, ^95^, ^96^ ^]^ Overall the nitride‐based materials possess excellent properties required for biosensing in substrate‐based biosensors as these materials can be integrated into different sensor platforms, including lab‐on‐chip devices, implantable sensors, and point‐of‐care diagnostic tools, contributing to advancements in healthcare, environmental monitoring, and food safety.

## 4 Chemiresistor‐Based Sensors and Related Challenges

2D material‐based field‐effect transistor chemiresistor biosensors represent a promising class of biosensing platforms with unique capabilities. These biosensors utilize the sensing mechanism of FETs combined with the chemiresistor principle, where changes in conductivity or resistance are detected upon interaction with target analytes. By integrating 2D materials such **as graphene**,^[^ ^97^, ^98^, ^99^, ^100^ ^]^ **transition metal dichalcogenides**,^[^ ^101^, ^102^, ^103^, ^104^ ^]^ or **black phosphorus** ^[^ ^105^, ^106^, ^107^, ^108^ ^]^ into the device architecture, these biosensors offer several advantages. First, the large surface area and atomically thin nature of 2D materials provide ample sites for biomolecule immobilization, enhancing the sensor's sensitivity. Second, the high carrier mobility and electrical properties of 2D materials facilitate rapid and precise detection of analyte‐induced changes in conductivity or resistance. Additionally, the tunable bandgap and surface functionalization capabilities of 2D materials enable selective detection of specific biomolecules.

**Figure 16:**
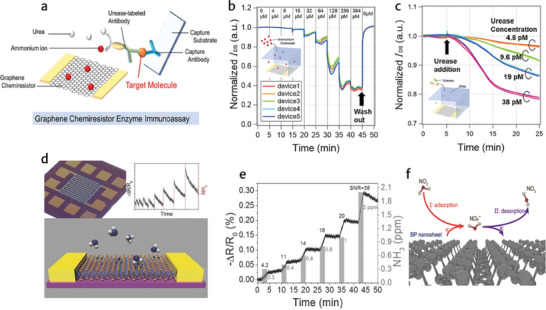
a) The schematic of a graphene chemiresistor FET device in which a patterned graphene sheet is bridged between source and drain electrodes. b) Graphene chemiresistor response to ammonium carbonate in an aqueous solution (*n* = 5). I_DS_ was normalized by the initial value. c) Graphene chemiresistor response to the urease reaction. The I_DS_ time course results for the three representative devices are displayed for each urease concentration. (a–c) Reproduced with permission.^[^ ^98^ ^]^ Copyright 2024, Elsevier. d) FET‐based on molybdenum disulfide (MoS_2_) grown by sulfurization of sputtered molybdenum layers. The results revealed the electrical conductivity of MoS_2_ films is highly sensitive to NH_3_ adsorption, consistent with n‐type semiconducting behavior. A sensitivity of 300 ppb at room temperature is achieved, showing the high potential of 2D transition metal‐dichalcogenides for sensing. e) The plot representing sensor response percentile resistance change versus time of the MoS_2_ film with a bias voltage of 0.5 V, upon consequent NH_3_ exposures at various concentrations ranging 300 ppb to 2 ppm. The gray vertical bars indicate NH_3_ gas injections for 2 min. The signal‐to‐noise ratio (SNR) of each folding curve is shown to verify a sensible signal. (d,e) Reproduced with permission.^[^ ^109^ ^]^ Copyright 2013, Wiley. f) Schematic of the NO_2_‐sensing using BP nanosheets. Reproduced with permission.^[^ ^107^ ^]^ Copyright 2020, American Chemical Society.

For instance, a most recent experimental investigation includes the development of a graphene chemiresistor for precise detection of Urease and other antigens. The main drawback of using FET‐based sensors is of capturing the small target molecules on graphene with sufficiently low ion concentrations. This has been overcome by the development of graphene chemiresistor enzyme immunoassay. Principally, in enzyme immunoassays, the detection of the target molecule and its concentration relies on detecting the reaction product produced by the enzyme‐labeled on the target molecule, rather than directly sensing the charge of the target molecule itself. To conduct an enzyme immunoassay effectively, it's crucial to select a suitable enzyme‐substrate pair, facilitating changes in electrical conductivity caused by the adsorption of the reaction product on the semiconductor channel. Naruto, et, al, utilized graphene, as it is known for its sensitivity to ammonium ions generated by urease reactions on its surface.^[^ ^98^, ^100^ ^]^ They utilized urease in enzyme immunoassay, catalyzing the hydrolysis of urea to carbon dioxide and ammonia, both expected to ionize in a neutral pH buffer. To label the target molecule, urease‐labeled antibodies were utilized. Traditionally, in enzyme immunoassays, separating labeled antibodies bound to the target molecule from those remaining free‐floating in the solution is essential (bound/free separation). In the described study, a solid‐liquid separation method was adopted by utilizing a substrate, immobilized with an antibody, was incubated in a solution containing the target molecule and urease‐labeled antibodies. This facilitated sandwich formation when the target molecules were present, a process conducted separately from the graphene sensors (**Figure** **16**a). After the formation of a FET biosensor, utilizing the enzyme immunoassay concept, the differences in the responses of the devices to urea and the products of the urease reaction, changes in I_DS_ (drain current) upon the introduction of either urea or ammonium carbonate were presented in Figure 16b. The graphene chemiresistor's I_DS_ was evaluated in a solution of 0.001 × PBS containing 0.2% Tween 20, with either urea or ammonium carbonate [(NH4)2CO3] added. The shifts in I_DS_ corresponding to the concentrations of urea or ammonium carbonate were examined. Moreover, the successful detection limit for urease was determined to be 4.8 pm through the urease reaction. With a urease concentration of 4.8 pm and an electrolyte volume of 250 µL, the electrolyte contains ≈7.2 × 10^8 urease molecules. In the graphene chemiresistor enzyme immunoassay, urease was introduced into the electrolyte using a capture substrate. Given that the area of the substrate immersed in the electrolyte is 100 mm^2^, the required urease density for sensor operation was reported to be 7.2 molecules µm^−2^. This density seems realistic considering the size of the protein, indicating that the graphene chemiresistor enzyme immunoassay could be a viable approach (see Figure 16c). Kangho et, al, examined the sensor performance of scalable MoS_2_ films synthesized via sulfurization of sputtered Mo thin films, which yielded highly uniform and structured MoS_2_ patterns. These patterns were directly accessible for electrode deposition using shadow masks. This two‐step process flow resulted in highly sensitive sensors capable of detecting ammonia (NH_3_) at sub‐parts‐per‐million (ppm) levels. The work demonstrated that layered 2D materials integrated seamlessly with semiconductor fabrication techniques, paving the way for cost‐effective, high‐performance devices as shown in Figure 16d. The experimental investigations depict typical gas sensor response curves across various concentrations of NH3 gas, ranging from 2 to 30 ppm, with a bias voltage of 0.5 V. NH_3_, acting as an electron donor, exhibits n‐doping characteristics. Upon exposure to gaseous NH_3_, adsorbed molecules on the MoS2 surface shift the Fermi level toward the conduction band, leading to a decrease in resistance consistent with n‐type behavior. The MoS_2_ film exhibits an immediate response, even with gases introduced for just 15 s. Notably, the response is constrained by the relatively large volume of the gas sensing chamber. The sensor functions effectively at sub‐ppm levels, as evident in Figure 16e, with a clear signal detectable down to 300 ppb. The sensor's sensitivity correlates linearly with the concentration of NH_3_ introduced within the low concentration range.^[^ ^109^ ^]^ In addition to the above, Tianding and coworkers reported a highly sensitive and selective chemiresistive nitrogen dioxide (NO_2_) sensor utilizing liquid‐phase‐exfoliated black phosphorus (BP) nanosheets. Their approach combines probe sonication and ice‐water bath sonication to produce BP nanosheets with controllable sizes. The schematic of the sensor is presented in Figure 16f. The characterization of the developed sensor reveals a significant sensing response of 88%, high selectivity, and complete recovery when exposed to 100 ppb NO_2_ gas at room temperature. Their proposed method enables the fabrication of nanosheets with adjustable gas‐sensing properties, facilitating their practical deployment in high‐performance NOx sensing.^[^ ^107^ ^]^

However, challenges such as material instability, device reproducibility, and biocompatibility need to be addressed to realize the full potential of 2D material‐based FET chemiresistor biosensors.^[^ ^102^, ^110^, ^111^, ^112^ ^]^ With continued research and development, these biosensors hold great promise for applications in healthcare, environmental monitoring, and biotechnology, offering sensitive, selective, and rapid detection of a wide range of biological and chemical analytes.

Such biosensing devices have the potential to open doors for the creation of novel biocompatible and transient biosensors for a variety of applications. **Table** **1** provides a detailed view of 2D‐based FET biosensors.

**Table 1:** Comparative Analysis of Recently Published 2D Materials‐Based Biosensors.

Sensor Type	Material	Analyte	Detection Limit	Mechanism	References
FET	MoS_2_	Streptavidin	1 fm	Electrostatic Doping	[113]
FET	Graphene	Exosomes	0.1 µg mL^−1^	Charge Sharing	[114]
FET	BP	IgG protein	5 pm ng mL^−1^	Electrostatic Gating	[115]
FET	WSe_2_	SARS‐CoV‐2 spike protein	25 fg µL^−1^	Doping/Charge transfer	[72]
FET	SnS_2_/h‐BN	Streptavidin	0.5 pm	Charge Sharing	[116]
FET	RGO	HepG2‐Mvs	4.8 pm	Gating Effect	[117]
FET	Graphene	Anthrax Toxin	120 fm	Charge transfer	[118]
FET	RGO	HPV‐16 E7	1.75 nm	Charge transfer	[119]
FET	Graphene	E.Coli	102 CFU ml^−1^	Electrostatic Gating	[120]
FET	Graphene	Insulin	35 pm	Gating Effect	[121]
FET	Graphene‐Au NPs	DNA aptamer	15 am	Electrostatic gating	[122]
FET	Graphene	IL‐6	618 fm	Charge sharing	[123]
FET	Graphene	IL‐6	8 pm	Charge sharing	[124]
FET	Graphene	Cytokine (IL‐6)	10.5 pm	Charge transfer	[125]
FET	Graphene	IFN‐γ	82.7 pm	Charge sharing/transfer	[126]
FET	Graphene	TNF‐α	2.75 pm	Electrostatic	[127]
FET	Graphene	Lysozyme	10 pm	Charge sharing/transfer	[128]
FET	WSe_2_	Glucose	1.0–10 mm	Charge sharing/transfer	[73]
FET	Graphene	Thrombin	2.6 pm	Gating Effect	[60]
FET	Graphene	HER2	0.1 pg mL^−1^	Gating Effect	[129]
FET	Graphene	OTA	10 pm	Electrostatic Gating	[130]
FET	MoS_2_	Cortisol	10^−18^ g mL^−1^	Charge sharing	[131]
FET	MoS_2_	cTn1	10 fm	Charge sharing‐	[132]
FET	MoS_2_	KAN	1.06 nm	Charge transfer	[133]
FET	MoS_2_	miRNA‐155	0.03 fm	Charge sharing/transfer	[134]
FET	In_2_O_3_	Glucose	0.1 mm	Protonation	[135]
FET	In_2_O_3_	Glucose	1 fm	Protonation	[81]
FET	Phosphorene	Alpha‐fetoprotein	0.1 ppb	Charge transfer	[83]
FET	BP	BP biocompatibility	6.25 µg mL^−1^	Gating effect	[84]
FET	BP	IgG protein	10 ng mL^−1^	Gating effect	[115]
FET	MoO_3_	BSA protein	250 µg mL^−1^	Charge transfer	[136]
FET	Graphene	Biotin	0.37 pm	Charge transfer	[137]
FET	2D‐Carbon	Pesticide	100 fm	Charge transfer	[138]
FET	Graphene	Procalcitonin	0.82 ag mL^−1^	Electrostatic potential change	[139]
FET	V_2_O_5_/GO_x_	Glucose	0.16 µmol L^−1^	Charge transfer	[140]
Chemiresistor	Graphene	Influenza virus nucleoprotein	100 pm	Charge transfer	[98]
Chemiresistor	Graphene	NO_2_ and NH_3_	15 and 160 ppb	Charge transfer	[97]
Chemiresistor	MoS_2_	TEA	35 ppm	Charge transfer	[101]
Chemiresistor	BP‐Ti_3_C_2_Tx	NO_2_	50 ppb	Charge transfer	[106]
Chemiresistor	MoSe_2_	NH_3_	50 ppm	Charge transfer	[103]

## 5 Biomedical Applications of FET Biosensors

### 5.1 Pathogenic Detection

2D materials‐based biosensors offer promising abilities for pathogen detection in biomedical applications. These biosensors allow for the rapid, sensitive, and specific detection of numerous diseases, including bacteria, viruses, and fungi, by utilizing the unique features of 2D materials such as graphene, transition metal dichalcogenides, and black phosphorus. The huge surface‐to‐volume ratio and strong electrical conductivity of 2D materials allow for the efficient immobilization of biomolecular recognition elements such as antibodies and aptamers, which improves pathogen detection sensitivity and selectivity. Furthermore, the flexibility and biocompatibility of 2D materials allow for their incorporation into miniaturized and wearable devices for point‐of‐care diagnostics and remote monitoring of infectious diseases. Furthermore, the adaptability of 2D materials allows for multiplexed detection of various pathogens in complicated biological samples, providing comprehensive and real‐time data for disease. In the fight against the pandemic, 2D materials‐based FET biosensors for COVID‐19 detection have represented a major advancement.

To detect the SARS‐CoV‐2 virus (COVID‐19), Seo, G., et al created a graphene‐based biosensing system in which the graphene channel material functionalized the SARS‐CoV‐2 antigen. Using antigen protein, cultured virus, and nasopharyngeal swab specimens from COVID‐19 patients, the sensor's functionality was demonstrated. Following pyrene butanoic acid succinimidyl ester (PBASE) functionalization, they evaluated the G‐FET transfer curves, which revealed a positive shift because of the pyrene group's p doping effect. The transfer curve was negatively altered, indicating that graphene was doped with n‐doping by the antibody's positive charge. To maintain viral vitality, this FET sensor could detect quantities of 100 fg/ml in clinical transport medium and 1 fg ml^−1^ in PBS. Additionally, the antibody's identity was verified using enzyme‐linked immunoassay (ELISA).^[^ ^44^ ^]^ Similarly, the creation and effective use of a laser‐induced graphene field‐effect transistor (LIG‐FET) for the quick and accurate identification of the SARS‐CoV‐2 virus, which causes COVID‐19, is described in another study by Cui, T.‐R., et al. The LIG‐FET has an oyster reef‐like porous graphene channel that is intended to improve the interface between the viral protein and the sensing area. It features different degrees of reduction of laser‐induced graphene. The FET exhibits extraordinary capabilities after antibodies are immobilized within the channel. It can detect the SARS‐CoV‐2 spike protein at concentrations as low as 1 pg/mL in phosphate‐buffered saline (PBS) and 1 ng mL^−1^ in human serum in ≈15 min. The sensor shows a noteworthy high selectivity for the spike protein (associated with SARS‐CoV‐2) and clears the path for a quick and inexpensive COVID‐19 detection technique.^[^ ^141^ ^]^ Moreover, graphene and reduced graphene oxide (rGO) have been studied in the FET‐based detection of bacteria and viruses.^[^ ^6^, ^142^, ^143^, ^144^ ^]^ Also, using MoS_2_/TiO_2_ hybrid nanostructure FET, Moudgil et al. have demonstrated highly selective and sensitive detection of gram‐positive bacteria.^[^ ^145^ ^]^

### 5.2 Point of Care Device

The Point of Care (POC) Device, as a biomedical application of FET‐based biosensors, holds substantial potential to revolutionize healthcare sectors by enabling rapid and accurate diagnosis at the point of need. These portable and user‐friendly devices integrate FET biosensors with microfluidic systems and advanced signal processing algorithms to detect biomarkers related with various diseases or health conditions directly from patient samples, such as blood, saliva, or urine. The real‐time detection capabilities of FET biosensors allow for timely diagnosis and monitoring of diseases, including infectious diseases, cancer, diabetes, and cardiovascular disorders.^[^ ^146^ ^]^ POC devices offer several advantages, including rapid turnaround time, minimal sample preparation, low cost, and the ability to perform tests outside of laboratory settings, making them valuable for resource‐limited or remote areas.^[^ ^147^, ^148^ ^]^

J. Scotto et al. introduced a gate‐field‐effect transistor (gFET) setup with a coplanar Ag/AgCl gate for real‐time monitoring of the kinetic adsorption of polyelectrolytes as charged macromolecules.^[^ ^149^ ^]^ This configuration enabled sequential adsorption processes to be measured without interrupting current measurement during Layer‐by‐Layer (LbL) assembly, making it suitable for point‐of‐care applications. Comparative monitoring using different techniques provided insights, revealing the device's ability to sense charges beyond the solution Debye length through a direct electrostatic mechanism, enabling real‐time monitoring even in high ionic strength conditions. Biasing the gFET through a constant gate potential increased sensitivity while maintaining kinetic response, enhancing its suitability for point‐of‐care diagnostics. Changes in current caused by polyelectrolyte layer adsorption decreased as film thickness increased, consistent with theoretical models. Overall, the study supports the use of gFET devices as miniaturized, fast, precise, and cost‐effective tools for real‐time monitoring and point‐of‐care diagnostics in biotechnology, particularly in high ionic strength environments.

### 5.3 Drug Discovery

2D materials‐based Field‐Effect Transistor (FET) devices offer promising avenues for drug discovery due to their unique properties and versatility. These devices can be tailored to detect various biomolecules involved in drug interactions with high sensitivity and specificity. By functionalizing the 2D materials' surfaces with specific receptors or ligands, FET biosensors can selectively capture target molecules, such as proteins, enzymes, or nucleic acids, relevant to drug discovery processes. The real‐time monitoring capability of FET devices allows for the dynamic observation of molecular interactions, providing valuable insights into drug‐receptor binding kinetics, affinity, and drug efficacy. Additionally, the miniaturized nature of 2D materials‐based FET devices enables high‐throughput screening of drug candidates, facilitating rapid and cost‐effective drug discovery workflows. Furthermore, these biosensors can be integrated into lab‐on‐a‐chip platforms, enabling point‐of‐care testing and personalized medicine applications.

S. Tsai et al. proposed a novel drug‐screening platform utilizing stretch‐out electrical double layer (EDL)‐gated field‐effect transistor‐based biosensors (BioFETs).^[^ ^150^ ^]^ These sensors effectively amplify electrophysiological signals from mammalian cardiomyocytes (H9c2). By employing a stretch‐out configuration, the researchers prevent chemical corrosion on FETs, thereby extending the lifespan of the BioFET system.

### 5.4 Other Bio‐Applications

#### 5.4.1 Detection of Biological Specimens

**Figure 17:**
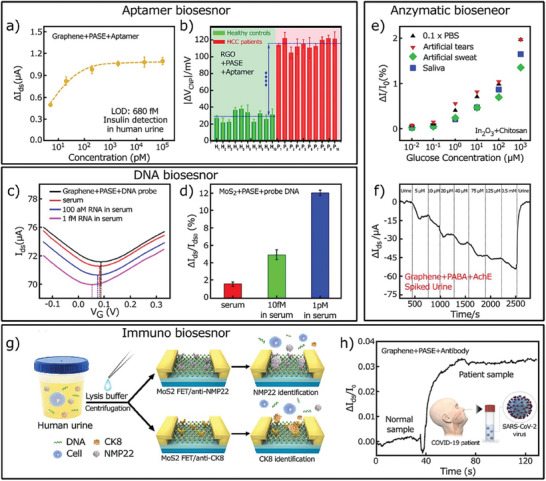
Findings from 2D‐based FET biosensors in clinical samples. a) Response of a GFET aptasensor to varying insulin concentrations in human urine. Reproduced with permission.^[^ ^123^ ^]^ Copyright 2020, American Chemical Society. b) Comparison of the RFET aptasensor response in ten healthy and hepatocellular carcinoma (HCC) patient samples using human serums. Reproduced with permission.^[^ ^117^ ^]^ Copyright 2020, American Chemical Society. c) Detection of Dirac voltage change in human serum containing 100 am and 1 fm target RNA using a graphene‐based DNA biosensor. Reproduced with permission.^[^ ^154^ ^]^ Copyright 2020, Elsevier. d) Reaction of the MoS_2_‐based DNA biosensor to two distinct target DNA concentrations in human serum. Reproduced with permission.^[^ ^155^ ^]^ Copyright 2018, Elsevier. e) Capacity of In_2_O_3_‐based enzymatic biosensors to detect glucose in artificial tears, saliva, and 0.1 x PBS solution. Reproduced with permission.^[^ ^156^ ^]^ Copyright 2018, American Chemical Society. f) Spiked‐Ach biotarget detection in human urine using an enzymatic biosensor based on graphene. Reproduced with permission.^[^ ^157^ ^]^ Copyright 2020, Elsevier. g) NMP22 and CK8 biotarget detection using a MoS_2_‐based FET immunosensor. Reproduced with permission.^[^ ^158^ ^]^ Copyright 2020, Springer. h) GFET immunosensor detection of the SARS‐CoV‐2 virus in a patient's diluted nasopharyngeal swab sample. Reproduced with permission.^[^ ^44^ ^]^ Copyright 2020, American Chemical Society.

The combination of Field‐Effect Transistors (FETs) with 2D materials have further applications and provide a wide range of opportunities for sensitive, selective, and real‐time detection in a variety of biological targets, such as sweat, serum, urine, and saliva. With diluted human urine samples, Hao et al. successfully measured insulin, demonstrating a 680 fm limit of detection (LOD).^[^ ^123^ ^]^ The graphene‐based FET (GFET) aptasensor's drain current fluctuates depending on the amount of insulin present in a person's urine. **Figure** **17**a shows this variation. The Dirac voltage shift for a reduced graphene oxide (RGO)‐based aptasensor is examined in Figure 17b between samples of human serum from healthy controls and hepatocellular carcinoma (HCC) patients.^[^ ^117^ ^]^ Its potential for illness diagnosis is highlighted by the detectable Dirac voltage shift observed in patient samples. Furthermore, the detection of biotargets in saliva has been accomplished with the use of 2D‐based FET aptasensors^[^ ^119^, ^125^, ^151^ ^]^ and sweat,^[^ ^152^, ^153^ ^]^ expanding their applicability beyond urine and serum. The majority of testing within the domain of 2D‐based FET DNA biosensors involves the use of serum samples from humans. Figure 17c illustrates a GFET DNA biosensor operating in fresh blood and serum, with target RNAs present at concentrations of 100 am and 1 fm.^[^ ^154^ ^]^ Mei et al. showed that serum with different cDNA concentrations may be used to test the responsiveness of an FET channel with MoS_2_ + probe DNA in Figure 17d.^[^ ^155^ ^]^ Enzymatic biosensors based on 2D FETs have undergone successful testing across diverse clinical samples, including tears, sweat, urine, serum, and saliva. Liu et al.^[^ ^156^ ^]^ employed an In2O3 layer to detect glucose in simulated tears, perspiration, and saliva. The sensing response of the FET in these three samples is depicted in Figure 17e, with a reference to 0.1 × PBS. Interestingly, saliva, perspiration, and tears can also detect glucose in lower quantities than blood.^[^ ^156^ ^]^ Acetylcholine (Ach) was spiked into a urine sample by Fenoy et al. to demonstrate the FET biosensor's responsiveness to different concentrations.^[^ ^157^ ^]^ The results are shown in Figure 17f, where higher signals are associated with rising Ach concentrations from 5 µm to 0.5 mm. As shown in the schematic representation in Figure 17g,^[^ ^158^ ^]^ Yang et al. identified bio‐based targets like NMP22 and CK8 as potential biomarkers to detect human bladder cancer in urine samples by using a functionalized MoS_2_ layer.

Seo et al. utilized a GFET immunosensor to detect the SARS‐CoV‐2 virus in diluted nasopharyngeal swab samples. Figure 17h illustrates the sensor response to both healthy and infected samples containing the virus. Additionally, other FET immunosensors based on 2D materials have undergone assessment in human serum samples. Chen et al. investigated various concentrations of the Ebola virus and monitored the current changes corresponding to each concentration.^[^ ^159^ ^]^ These FET biosensors demonstrate high accuracy and selectivity in identifying biotargets within authentic human samples. As promising point‐of‐care (PoC) devices for contemporary medicine, they hold the potential for diagnosing or monitoring diseases in real‐world scenarios.

#### 5.4.2 Environmental Monitoring

Field‐effect transistor (FET) biosensors play an essential role in environmental monitoring by detecting various environmental pollutants and contaminants. These biosensors are capable of sensing a wide range of analytes, containing heavy metals, pesticides, organic pollutants, and toxins, present in air, water, and soil. The sensing mechanism utilizes the immobilization of specific receptors or biomolecules onto the FET surface, which selectively interacts with the target analyte, leading to a change in the electrical properties of the FET devices. This change is then transduced into an electrical signal, providing real‐time and sensitive detection of the target analyte as a pollutant. FET biosensors offer advantages such as high sensitivity, speedy response time, portability, and ease of integration into monitoring systems, making them a valuable tool for environmental monitoring applications.

#### 5.4.3 Biosecurity Applications

Field‐effect transistor (FET) devices are employed in biosecurity applications to monitor and identify hazardous materials, infections, and biological threats in order to protect public health and safety. Biological agents that are hazardous to human health and the environment, such as bacteria, viruses, and toxins, can be quickly and accurately detected with biosensors.^[^ ^160^ ^]^ These biosensors use certain biomolecules or receptors that interact with target biological substances in a targeted manner, changing their electrical characteristics in a measured way. FET biosensors can offer early warning of possible biosecurity hazards by identifying these changes, allowing for prompt responses and preventive measures. Furthermore, FET devices can be included in surveillance systems to monitor high‐risk locations, transportation hubs, and vital infrastructure continuously, improving biosecurity and readiness against infectious diseases and bioterrorism.

#### 5.4.4 Water Quality Monitoring

The Field‐Effect Transistors composed of 2D materials like graphene have enabled the creation of biosensors for monitoring water quality. These biosensors detect toxins, diseases, and pollutants in water sources in a sensitive, quick, and real‐time manner. FET‐based biosensors use the unique features of 2D materials like graphene and transition metal dichalcogenides to detect trace quantities of pollutants, heavy metals, and microbiological contaminants in water samples. This application has enormous promise for assuring the safety and security of drinking water sources, as well as environmental sustainability.

A. Maity et al. employed wet transfer techniques, impedance and noise measurements, and machine learning algorithms to streamline the fabrication process of graphene‐based field‐effect transistor (GFET) sensor arrays and accurately identify faulty devices.^[^ ^161^ ^]^ Our sensors demonstrated real‐time detection capabilities for heavy‐metal ions (lead and mercury) as well as *E. coli* bacteria in flowing tap water. This research provides a robust quality control protocol, enhancing the scalability and reliability of electronic sensors for monitoring pollutants in dynamic water systems.

### 5.5 FET‐Based Wearable Biosensors

The advancement of wearable biosensors is significantly dependent on the development of extremely flexible, mobile, and persistent power sources, which are required for sensing functionality in wearable bio‐electronics.^[^ ^162^ ^]^ 2D materials are becoming more and more appealing in the field of flexible wearable device development because of their exceptional physical qualities and ultra‐low thickness. The wearable biosensors based on various materials are described in this section.

**Figure 18:**
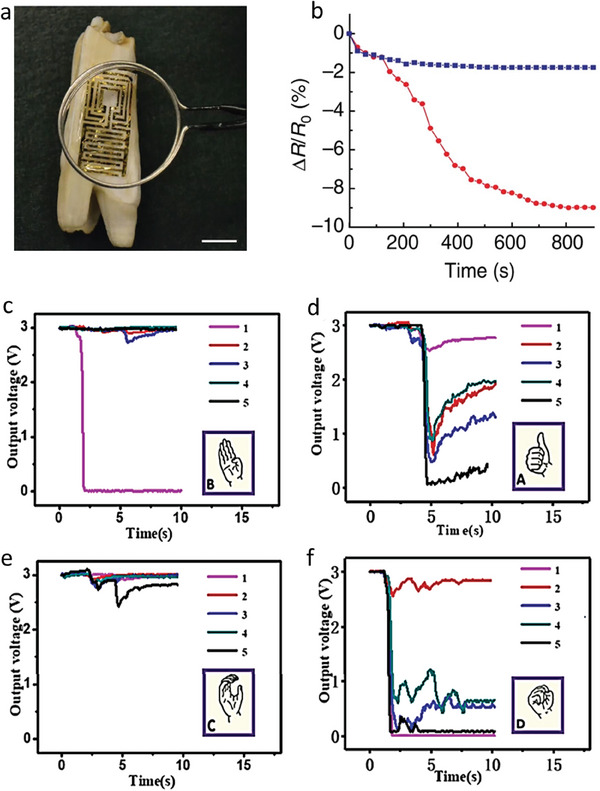
2D materials are used in wearable sensors. a,b) A graphene dental patch sensor exposed to ≈100 H. pylori cells in human saliva over time displays the % change in resistance (red line). A blue line represents the response to a “blank” saliva solution. (a,b) Reproduced with permission.^[^ ^163^ ^]^ Copyright 2012, Nature. c–f) The device is designed to capture sign language gestures and is fitted with five strain sensors. Each sensor corresponds to a specific finger and allows the identification of sign language gestures “A”, “B”, “C”, and “D”. Fingers are labeled as follows: thumb, index finger, middle finger, ring finger, and little finger. (c–f) Reproduced with permission.^[^ ^164^ ^]^ 2021, Springer.

Mannoor et al. reported on a novel technique for creating wireless graphene nano‐sensors on biomaterials via silk biosorption,^[^ ^163^ ^]^ as shown in **Figure** **18**a, and its response is recorded in Figure 18b. By exhaling onto the tooth, the integration of this sensor grants remote breath monitoring. The study also explores how the sensor responds to H. pylori, a harmful bacteria that is common in saliva and the stomach and is linked to conditions including cancer and ulcers. The sensor, which is based on antimicrobial peptides (AMPs), selectively recognizes and binds to H. pylori cells in human saliva. Real‐time changes in graphene resistance are found after contact to saliva containing ≈100 H. pylori cells. This detection approach determines a linear relationship between bacterial concentration and resistance change, with a detection limit of ≈100 cells, which is far lower than conventional techniques. Moreover, this work underlines the integration of biosensors into wearable technologies. The integration of such sensors into ordinary goods, such as tooth sensors, allows for nonstop and efficient health monitoring. This breakthrough is capable of early disease detection and tailored healthcare management, leading to a new era of wearable biosensor devices. Furthermore, the graphene nano‐sensors are printed onto water‐soluble silk thin‐film substrates, and interdigitated electrodes are then applied. After that, the hybrid structure of graphene, electrode, and silk is applied to biomaterials like tissue or tooth enamel. Highly sensitive chemical and biological sensing is made possible by this inventive gadget architecture, which can detect as few as one bacterium. Additionally, the system accomplishes wireless remote charging and readout, which represents a noteworthy breakthrough in the field of oral health sensor technology.^[^ ^163^ ^]^

In another study, a glove system designed specifically for identifying gestures in sign language is presented by Yuan et al.^[^ ^164^ ^]^ The system consists of an artificial neural network algorithm, a multi‐channel data collecting module, and flexible cross‐reticulated graphene (CRG) sensors. Because of the acquisition module's multiple‐channel design, signals from five sensors can be collected and sent to an intelligent terminal so that the hand gesture detection algorithm may be run. The recognition technique granted high‐level accuracy, with the neural network attaining recognition rates of more than 90% for the 22 letters of the English alphabet. Overall, the system's accuracy in recognizing hand movements exceeds 86%. This study shows the potential of CRG flexible sensors and neural network algorithms in constructing efficient and effective gesture recognition systems, which has implications for developing communication accessibility for sign language users.

Figure 18c–f demonstrates the voltage changes recorded by these sensors during the gestures “A”, “B”, “C”, and “D”. These figures show that these four movements have distinct voltage patterns, making them easy to recognize even without specialized equipment. To test the stability of gesture recognition, the gesture “A” was repeated 16 times, and the resulting voltage variations were evaluated. The findings show that, despite modest differences between repeats, gesture “A” is continuously recognizable. However, as the study expands to include all 26 motions, the presence of noise becomes more noticeable due to the similarity of some movements. This noise makes it difficult to discriminate between motions, especially when several gestures have similar properties.

The developments in wearable biosensor systems are astonishing, especially the way that 2D materials are being incorporated to overcome the difficulties of creating microminiaturized sensors. Because of their remarkable mechanical, optical, and electrical qualities, 2D materials present a viable option for the development of creative and effective wearable biosensors. Recent advancements in this field of study demonstrate the potential use and diversity of 2D materials in wearable technology, with applications including diabetic patches, wearable wristbands, e‐skin, and contact lens sensors.^[^ ^165^ ^]^

## 6 Significance, Challenges, and Prospects

**Figure 19:**
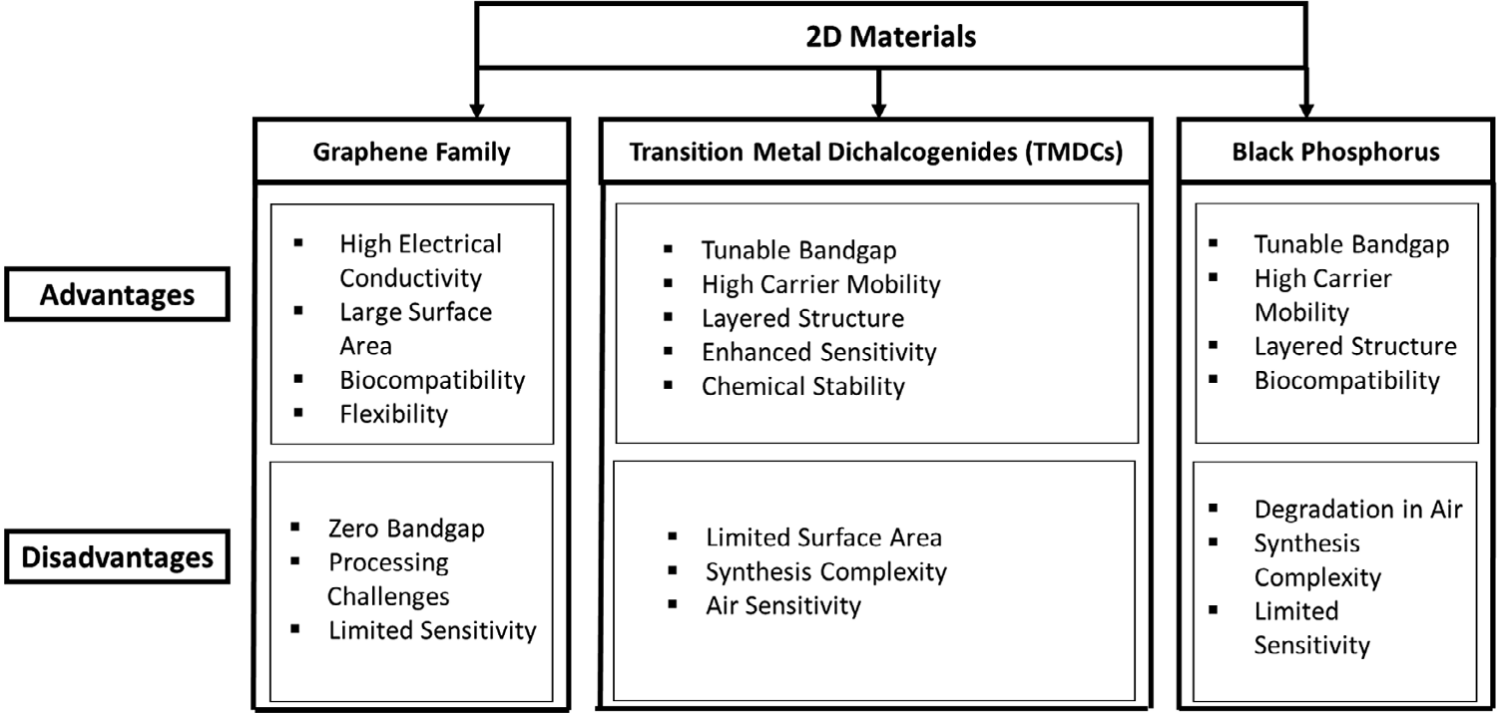
Assessment of the benefits and difficulties of using 2D materials as channel materials for FET biosensor applications.

2D FET biosensors have emerged as cutting‐edge technology with substantial implications for a variety of technical and scientific applications. The capacity of these biosensors to provide extremely sensitive and selective biomolecule detection makes them indispensable instruments in domains including biosecurity, environmental monitoring, and medical diagnostics.^[^ ^166^, ^167^ ^]^ The better performance of 2D‐based FET biosensors over conventional biosensing platforms emphasizes their significance. The remarkable electrical characteristics of graphene and other 2D materials enable improved signal transduction and effective biological analyte detection. Because 2D materials have a greater surface area, there is more room for immobilizing biomolecules, which leads to a better binding capacity and, ultimately, improved sensitivity.^[^ ^168^ ^]^ Because these biosensors can identify traces of biomarkers, they are essential for early illness detection and surveillance. 2D‐based FET biosensors have a lot of potential for the quick and precise diagnosis of such illnesses as cancer, diabetes, and infectious diseases in medical applications. These biosensors' speed and accuracy allow for early intervention and individualized treatment plans, which can have a substantial impact on patient outcomes.^[^ ^169^, ^170^ ^]^ The widespread use of 2D‐based FET biosensors is not without difficulties, despite their enormous potential. The reproducibility of the device fabrication process is one significant obstacle. It is necessary to precisely manage the material characteristics, layer thickness, and surface functionalization to synthesize and integrate 2D materials into biosensor systems. To ensure the dependability and repeatability of sensor performance, a crucial component in transferring these technologies from the lab to real‐world applications (commercialization) must be achieved. Optimizing sensitivity and selectivity presents another difficulty. Even though 2D materials have great sensitivity, it is important to make sure the biosensor is selective for certain biomolecules. The precision of identification may be jeopardized by interference from intricate sample matrices or cross‐reactivity with related chemicals. Innovative approaches to surface functionalization and the creation of sophisticated recognition elements are needed to meet these challenges. Prospects for 2D‐based FET biosensors are bright, as continuing research endeavors to surmount present constraints and open novel avenues. It is anticipated that developments in materials science and nanotechnology will produce new 2D materials with customized characteristics, thereby improving sensor performance. Furthermore, scalability and affordability of these biosensors will probably be enhanced by advancements in microfabrication techniques, opening the door for their incorporation into point‐of‐care systems. Enhancing the analytical capabilities of 2D‐based FET biosensors is expected to be largely dependent on the incorporation of artificial intelligence and machine learning algorithms. By supporting real‐time data processing, pattern recognition, and the detection of minute variations in biosensor signals, these technologies can improve the precision and dependability of diagnoses. **Figure** **19** presents a schematic diagram evaluating the advantages and challenges of using 2D materials as channel materials for FET biosensor applications.^[^ ^165^ ^]^

To sum up, 2D‐based FET biosensors are a ground‐breaking development in biosensing technology that is highly significant, especially in the areas of environmental monitoring and healthcare. Although reproducibility, commercialization, and optimization remain obstacles, current research and technology developments should help to resolve these problems, creating exciting new opportunities for biosensor applications in the future. With their unparalleled sensitivity and selectivity in biomolecule detection, 2D‐based FET biosensors are expected to have a substantial and long‐lasting influence on a multitude of industries as the area continues to advance.

## 7 Drawbacks

Graphene‐based field‐effect transistor (FET) biosensors offer remarkable sensitivity and rapid response times, making them favorable candidates for various biosensing applications. However, they also present several drawbacks that researchers need to address for practical applications. One significant challenge is the limited surface area available for biomolecule immobilization due to graphene's atomically thin nature, potentially restricting the binding capacity and affecting the sensor's sensitivity. Additionally, graphene's lack of an inherent bandgap poses hurdles in achieving high on/off ratios, leading to higher noise levels and reduced signal‐to‐noise ratios in biosensing applications. Another concern is the stability of graphene‐based FET biosensors, as graphene is susceptible to environmental factors such as humidity and oxygen exposure, which can degrade device performance over the passage of time. Moreover, the scalability and reproducibility of graphene synthesis and device fabrication processes remain challenging, hindering large‐scale production and commercialization.

Furthermore, for the TMDC‐based field‐effect transistor (FET) biosensors, the major limitation is the synthesis and integration challenges associated with TMDC materials. Achieving high‐quality and uniform TMDC layers, especially over large areas, remains a complex and costly process, hindering scalability and mass production. Additionally, TMDC‐based FET biosensors often suffer from low carrier mobility and poor on/off ratios compared to graphene‐based counterparts, leading to reduced sensitivity and signal‐to‐noise ratios. Furthermore, TMDC materials are also vulnerable to environmental factors such as moisture and oxygen exposure, which can degrade device performance over time and limit their long‐term stability. Another drawback is the limited diversity of TMDC materials available, constraining the range of biomolecules that can be detected and the versatility of biosensor applications.

Furthermore, the major drawback associated with the extensive adoption of Black phosphorus‐based field‐effect transistor (FET) biosensors includes the inherent instability of black phosphorus in ambient conditions, particularly in the presence of oxygen and moisture, leading to degradation of device performance over time. This instability sets challenges for long‐term sensor operation and reliability, necessitating protective encapsulation or passivation strategies. Additionally, black phosphorus‐based FET biosensors may experience from poor device‐to‐device reproducibility due to variations in material quality and device fabrication processes. Achieving uniformity and consistency in black phosphorus‐based devices remains a challenge, hindering scalability and mass production. Furthermore, the limited understanding of black phosphorus's biocompatibility and potential cytotoxicity develops concerns about its use in biological applications.

Addressing these drawbacks requires innovative solutions in material synthesis techniques, surface functionalization techniques, device design, material engineering, and fabrication methods to enhance the sensor performance, stability, and manufacturability of these 2D‐based FET biosensors for practical biosensing applications.

## 8 Industrialization Prospects

2D materials such as graphene, transition metal dichalcogenides, and black phosphorus offer outstanding performance characteristics due to their unique electronic properties, high surface‐to‐volume ratios, and biocompatibility.^[^ ^171^ ^]^ Hence, 2D material‐based field‐effect transistor (FET) biosensors demonstrate promising potential for various industrial applications. In terms of performance, they offer high sensitivity, rapid response times, and excellent selectivity, making them ideal for detecting biomolecules with high precision. Additionally, their compatibility with miniaturization and integration into existing electronic devices further enhances their attractiveness for industrial use. Moreover, the tunable electronic properties of 2D materials allow for useful sensor designs tailored to specific biomolecular targets, enhancing their performance further.^[^ ^172^, ^173^ ^]^ However, despite their promising performance, the industrialization prospects of 2D material‐based FET biosensors may face challenges related to cost.

The scalability of synthesis techniques, material integrity, and device fabrication processes significantly influence the overall cost of 2D material‐based biosensors. Additionally, integration with existing production infrastructure and compatibility with mass production methods are critical factors impacting cost‐effectiveness. Although, it's crucial to assess the exact cost‐effectiveness of mass‐producing these sensors. While the initial fabrication costs may be relatively high due to the specially designed equipment and materials required, advancements in manufacturing techniques and economies of scale could drive down costs over time.^[^ ^174^ ^]^ Therefore, despite the current challenges, the growing demand for sensitive and reliable biosensing technologies suggests a bright industrial future for 2D material‐based FET biosensors.

## 9 Conclusion

In this comprehensive review, we discuss the emerging field of electrical devices based on 2D materials for biosensing applications, focusing on their enormous potential for reshaping the fields of diagnostics and healthcare. With the growing demand for quick, sensitive, and portable biosensors in a variety of industries, the development of new materials and unique sensor designs has become critical.

Our review critically investigates the working mechanisms and detection capabilities of biosensing devices employing graphene and various other 2D materials including TMDCs semiconductors. Their incorporation into FET biosensors brings forth advantages such as low detection limits (LOD), versatility, real‐time monitoring, label‐free diagnosis, and remarkable selectivity. The creation of novel point‐of‐care diagnostic instruments that are appropriate for telemedicine in the future is promised by the 2D‐based bio‐FETs. Additionally, a top‐down production method that is more in line with existing technology is typically linked to 2D bio‐FETs. Additionally, we also explained the diverse applications, ranging from conventional biosensors to the emerging frontier of wearable biosensors, emphasizing the versatility of 2D material‐based FET devices. By explaining the underlying principles governing their operation and discussing their advantages over conventional biosensors, we highlight the transformative potential of 2D materials in advancing the field of biosensing.

Furthermore, we provide an in‐depth assessment of the limitations and challenges these devices face, alongside the prospects and advancements on the horizon. By acknowledging these challenges, we pave the way for the future enhancement of biosensor technology. Furthermore, our investigation extends to prospects, providing an outlook on the evolving context of biosensors, and emphasizing the opportunities for innovation and development in the industry.

Of particular significance is the detailed comparison of field‐effect transistor (FET)–based biosensors, a key focus of our review, which is tabulated beside various other types of biosensors and their working mechanisms. This comparative analysis offers valuable awareness of the performance metrics and suitability of different biosensing platforms for diverse applications. Our review aims to promote more research and innovation in this fascinating subject while also educating the scientific community about the latest advancements in 2D materials‐based biosensors. The potential of advanced biosensor devices and their integration into everyday life further emphasizes the dynamic trajectory of biosensing technologies.

## Conflict of Interest

The authors declare no conflict of interest.

## Author Contributions

S.N. and G.D. contributed equally to this work. The project was designed and directed by G.D., while G.D. and S.N., provided the essential insights to collect data, techniques, and article content and wrote the review article. Z.M.S., M.W.Z., and A. R. were involved to collect the data and in reviewing the concluding narrative, critically analyzing the literature, and contributing to both the drafting and revision of the manuscript. D.K.K., A.I., and A.R.C. provide the resources and funds support for this research. M.Z.I., I.R., and K.H., also assisted to revise the manuscript. All authors actively participated in the literature review, data collection, and text revision.
